# Microglia Gravitate toward Amyloid Plaques Surrounded by Externalized Phosphatidylserine via TREM2

**DOI:** 10.1002/advs.202400064

**Published:** 2024-07-09

**Authors:** Jong‐Chan Park, Jong Won Han, Woochan Lee, Jieun Kim, Sang‐Eun Lee, Dongjoon Lee, Hayoung Choi, Jihui Han, You Jung Kang, Yen N. Diep, Hansang Cho, Rian Kang, Won Jong Yu, Jean Lee, Murim Choi, Sun‐Wha Im, Jong‐Il Kim, Inhee Mook‐Jung

**Affiliations:** ^1^ Department of Biophysics Sungkyunkwan University Suwon 16419 Republic of Korea; ^2^ Institute of Quantum Biophysics Sungkyunkwan University Suwon 16419 Republic of Korea; ^3^ Department of Metabiohealth Sungkyunkwan University Suwon 16419 Republic of Korea; ^4^ Department of Biochemistry and Biomedical Sciences College of Medicine Seoul National University Seoul 03080 Republic of Korea; ^5^ Genome Medicine Institute Medical Research Center Seoul National University Seoul 03080 Republic of Korea; ^6^ Department of Physiology and Biomedical Sciences College of Medicine Seoul National University Seoul 03080 Republic of Korea; ^7^ BK21 FOUR Biomedical Science Program College of Medicine Seoul National University Seoul 03080 Republic of Korea; ^8^ UK Dementia Research Institute Institute of Neurology University College London Gower Street London WC1E 6BT UK; ^9^ Neuroscience Research Institute Seoul National University Medical Research Center Seoul 03080 Republic of Korea; ^10^ Department of Intelligent Precision Healthcare Convergence Sungkyunkwan University Suwon 16419 Republic of Korea; ^11^ Department of Biomedical Sciences College of Medicine Seoul National University Seoul 03080 Republic of Korea; ^12^ Department of Biochemistry and Molecular Biology Kangwon National University School of Medicine, Gangwon Seoul 24341 Republic of Korea; ^13^ Cancer Research Institute Seoul National University Seoul 03080 Republic of Korea; ^14^ Department of Biochemistry and Molecular Biology Seoul National University College of Medicine Seoul 03080 Republic of Korea; ^15^ Convergence Dementia Research Center College of Medicine Seoul National University Seoul 03080 Republic of Korea

**Keywords:** Alzheimer's disease, beta‐amyloid, microglia, phosphatidylserine, TREM2

## Abstract

Microglia play a crucial role in synaptic elimination by engulfing dystrophic neurons via triggering receptors expressed on myeloid cells 2 (TREM2). They are also involved in the clearance of beta‐amyloid (Aβ) plaques in Alzheimer's disease (AD); nonetheless, the driving force behind TREM2‐mediated phagocytosis of beta‐amyloid (Aβ) plaques remains unknown. Here, using advanced 2D/3D/4D co‐culture systems with loss‐of‐function mutations in TREM2 (a frameshift mutation engineered in exon 2) brain organoids/microglia/assembloids, it is identified that the clearance of Aβ via TREM2 is accelerated by externalized phosphatidylserine (ePtdSer) generated from dystrophic neurons surrounding the Aβ plaques. Moreover, it is investigated whether microglia from both sporadic (CRISPR‐Cas9‐based *APOE4* lines) and familial (*APP^NL‐G‐F^*/*MAPT* double knock‐in mice) AD models show reduced levels of TREM2 and lack of phagocytic activity toward ePtdSer‐positive Aβ plaques. Herein new insight is provided into TREM2‐dependent microglial phagocytosis of Aβ plaques in the context of the presence of ePtdSer during AD progression.

## Graphical abstract

This study, utilizing advanced co‐culture models, shows that ePtdSer in dystrophic neurons surrounding Aβ plaques accelerates TREM2‐mediated Aβ phagocytosis. Additionally, it is demonstrated that microglia in an AD model have reduced TREM2 levels and lower phagocytic activity against ePtdSer‐positive Aβ plaques. These results offer new insights into TREM2‐dependent microglial phagocytosis of Aβ plaques in the context of ePtdSer during AD progression.

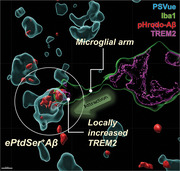

## 1 Introduction

Microglia are resident immune cells and one of the major types of neuroglia in the brain and spinal cord.^[^ ^1^ ^]^ They are necessary for normal brain function and also act as essential neuroprotectors, as they play a role in eliminating supernumerary synapses^[^ ^2^ ^]^ and clearance of misfolded proteins such as beta‐amyloid (Aβ) and neurofibrillary tangles in the brain.^[^ ^3^ ^]^ Recent studies have shown that microglia detect neuronal “eat me” signals, comprising externalized phosphatidylserine (ePtdSer) that are exposed on the surface of neurons, for the process of synapse pruning.^[^ ^4^ ^]^ Interestingly, Scott‐Hewitt and Rueda‐Carrasco *et al.* revealed that ePtdSer‐labeled synapse elimination is modulated by triggering receptor expressed on myeloid cells 2 (TREM2), which is a major phagocytic receptor of microglial cells.^[^ ^4^, ^5^ ^]^ They identified that synaptic elimination can be prevented through microglial loss of function of TREM2, thereby reducing the engulfment of ePtdSer^+^ synapses by microglial cells. These results imply that TREM2‐mediated phagocytosis can be associated with the presence of ePtdSer beyond the normal processes of synapse pruning because ePtdSer also can be exposed on damaged neurons or glial cells during the progression of neurological diseases as well.^[^ ^5^, ^6^ ^]^ However, the role of ePtdSer in the injured brain during the progression of diseases remains unclear; therefore, TREM2‐mediated phagocytosis in the vicinity of brain lesions such as Aβ plaques in the brains of the patients with Alzheimer's disease (AD) should be studied in the context of the presence of ePtdSer.

Recently, Huang *et al.* reported a noteworthy phenomenon wherein ePtdSer is found in the areas surrounding Aβ plaques in the brains of *APP/PS1* mice and speculated that ePtdSer‐associated Aβ plaques (ePtdSer^+^‐Aβs) may be a trace of the dystrophic neurites from damaged neurons.^[^ ^7^ ^]^ Since they mainly addressed Tyro3‐Axl‐Mer (TAM) receptor‐mediated microglial phagocytosis,^[^ ^8^ ^]^ however, they did not elaborate on the formation of ePtdSer^+^‐Aβs and their source of origin. Moreover, they did not comment on the relevance of ePtdSer^+^‐Aβs in TREM2‐mediated phagocytosis by microglial cells, although the colocalization of TREM2 with ePtdSer^+^‐Aβs has been observed.^[^ ^8^ ^]^ Thus, whether local ePtdSer expression informs TREM2‐mediated microglial Aβ uptake and gravitation of microglial cells toward Aβ plaques remains unclear.

Herein, we hypothesized that microglia exhibit TREM2‐dependent behavior toward ePtdSer^+^‐Aβs, which are generated in the vicinity of dystrophic neurons and Aβ plaques. Using induced pluripotent stem cell (iPSC)‐ derived neurons and 3D brain assembloids, we revealed that ePtdSer^+^‐Aβs are generated from dystrophic neurites of damaged neurons. Additionally, clustered regularly interspaced short palindromic repeats (CRISPR)‐Cas9‐based *TREM2/APOE* isogenic iPSC‐derived human AD microglial cells in 2D co‐culture models or 3D brain assembloids were used for high‐resolution or live‐cell imaging and 3D graphical rendering to identify the direct association between TREM2 and ePtdSer^+^‐Aβs.^[^ ^9^ ^]^ Finally, whether both sporadic (CRISPR‐Cas9‐based *APOE4*) and familial (*APP^NL‐G‐F^*/*MAPT* double knock‐in (dKI)) mice AD models exhibit a lack of TREM2‐mediated phagocytic activity toward ePtdSer^+^‐Aβs was investigated.^[^ ^10^ ^]^ We provide new insight into microglial TREM2‐dependent phagocytosis associated with PtdSer toward amyloid plaques in the brain.

## 2 Results

### 2.1 Experimental Design

**Figure 1:**
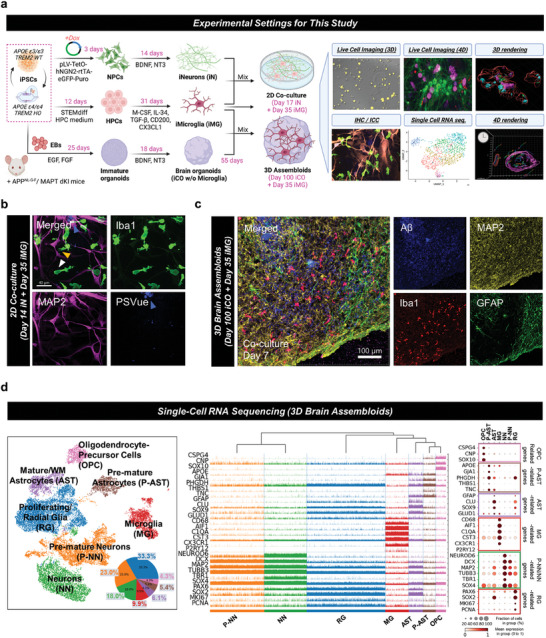
Experimental models (2D/3D/4D co‐culture) used in this study. a) Development of 2D/3D/4D co‐culture systems. The iPSC‐derived neurons (DIV 14) and microglia (DIV 35) were co‐cultured for immunocytochemistry, live cell imaging, and IMARIS 3D/4D rendering. Additionally, brain assembloids (DIV 100 brain organoids including DIV 35 iPSC‐derived microglia) were used for single‐cell RNA sequencing, immunohistochemistry, and IMARIS 3D rendering. b) Confocal microscopy imaging was used for the validation of 2D co‐culture systems. Human iPSC‐derived microglia (anti‐Iba1) mingle with iPSC‐derived neurons (anti‐MAP2), and externalized PtdSer (PSVue) are observed around the neurons. Green, microglia, anti‐Iba1; Pink, neurons, MAP2; Blue and white arrows, ePtdSer+, PSVue. Scale bar = 40 µm. c) Infiltration of microglia into the brain organoid. Blue, amyloid‐beta, anti‐D54D2; green, astrocytes, anti‐GFAP; red, microglia, anti‐Iba1; yellow, neurons, anti‐MAP2; white arrows, the deepest reaches of microglia. Scale bar = 100 µm. d) Characterization of brain assembloids via single‐cell RNA sequencing analysis. Uniform Manifold Approximation and Projection (UMAP) displaying the assembloids (n = 17,472 cells). A microglial population (a circle with a dotted line) was detected in the brain assembloids. Tracksplot showed gene expression levels by height. Dot plot showing the expression levels and fractions of cells expressing each cell type marker genes.

To demonstrate physiological interactions between neurons and microglia, 2D/3D co‐culture systems were established in various combinations with iPSC‐derived neurons (iNeurons), iPSC‐derived microglia (iMG), and iPSC‐derived brain organoids (iCOs) (**Figure** **1**a).^[^ ^9^, ^11^ ^]^ Along with these various culture methods, the AD mouse model (*APP^NL‐G‐F^*/*MAPT* dKI mice) was used.^[^ ^12^ ^]^ For visualization and quantification, multiple imaging techniques such as 3D live‐cell imaging (long‐term timelapse 2D imaging), 4D live‐cell imaging (3D confocal imaging with short‐term timelapse), and 3D surface‐rendering technology were used. For example, ePtdSer (stained using PSVue dye), swimming and phagocytic iMG, and interaction between iNeurons and iMG were captured using the 2D co‐culture model (Figure 1b). Validation of microglial phagocytic activity was conducted using Fluoresbrite YG microsphere (Polysciences, cat: 17147; Warrington, PA, USA), and phagocytosing microglia were captured and visualized using long‐term timelapse 2D imaging (Video S1, Supporting Information). For the advanced 3D disease model, iPSC‐derived brain assembloids (3D mixed‐culture models including both iCOs and iMG) were developed and validated through immunohistochemistry (IHC) (Figure 1c) and characterized using single‐cell RNA sequencing (scRNAseq) (Figure 1d). Microglial population was observed inside of the brain assembloids as well as neurons, pre‐mature neurons, astrocytes, pre‐mature astrocytes, oligodendrocyte precursor cells (OPCs), and proliferating/radial glial cells (Figure 1d). Constituent cells of the brain assembloids were further validated using the corresponding genes (e.g., *MAP2* for neurons, *GFAP* for astrocytes and glial cells, *TREM2* or *LPL* for microglia, and *CSPG4* for OPCs, etc.) (Figure 1c,d). Detailed methods and validations for the co‐culture systems were described in the Methods and Figures S1–S4 (Supporting Information). Moreover, we compared microglial‐specific genes between in iMG, 2D co‐cultured iMG, and 3D co‐cultured iMG (Figure S5, Supporting Information). As we expected, microglial genes with 3D showed relatively higher expression compared to the iMG‐only or 2D coculture model.

### 2.2 Phosphatidylserine (PtdSer) Improves Microglial Phagocytosis toward Aβ

**Figure 2:**
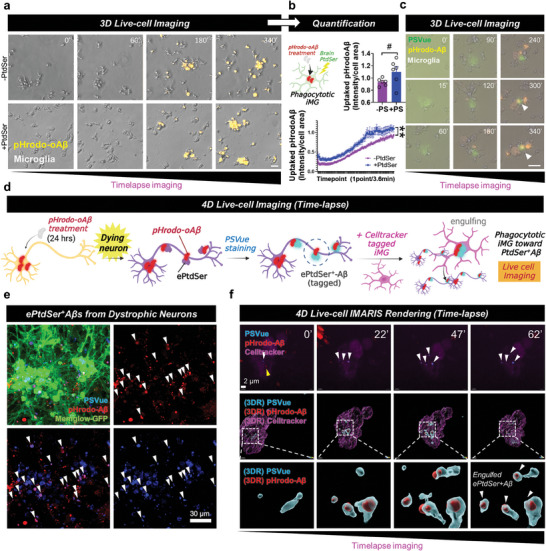
PtdSer improves microglial uptake of Aβ. a,b) Timelapse imaging of microglial uptake of pHrodo‐Aβs with/without PtdSer. Yellow, pHrodo‐Aβ. N = 6 slides for each group; average n = 27.68 cells were tracked per one field of view (scale bar = 20 µm). **p < 0.01, comparison of area under curves; ^#^ *p* < 0.1, independent *t*‐test. c) PtdSer initializes microglial uptake of pHrodo‐Aβs. Yellow, uptaken pHrodo‐Aβ; green, PSVue. Scale bar = 20 µm. d) Concept of 4D live‐cell imaging to visualize the uptake of ePtdSer^+^ Aβs by microglia. e) Generation of ePtdSer^+^ Aβs by dystrophic neurons. Green, Memglow‐GFP, neurons; blue, PSVue; red, pHrodo‐Aβ. f) 4D time‐lapse imaging with 3D rendering using IMARIS software. The internalized pHrodo‐Aβs were colocalized with ePtdSer (ePtdSer^+^ Aβs). White arrows, internalized ePtdSer^+^ Aβs; white box, 3D rendering position; blue, PSVue; red, pHrodo‐Aβs; pink, cell‐tracker 647, microglia.

Since it is well known that microglia engulf both ePtdSer^+^ synapses and Aβs,^[^ ^4^, ^13^ ^]^ we thought that the relationship between PtdSer and Aβa should be examined. Interestingly, iMG showed significantly better phagocytic performance toward pHrodo‐tagged oligomeric Aβ (pHrodo‐oAβ) when PtdSer was also treated rather than pHrodo‐oAβ alone (**Figure** **2**a,b, Video S2, Supporting Information). To investigate how PtdSer accelerated the uptake of oAβ, microglial cells were incubated with PtdSer‐specific dye (PSVue), pHrodo‐oAβ, and PtdSer. Long‐term timelapse imaging revealed that PSVue signals increased suggesting initiated uptake of pHrodo‐oAβ (Figure 2c, Video S3, Supporting Information). These findings indicate that PtdSer recognition can be a trigger for the microglial uptake of oAβ. For in‐depth visualization and better physiological conditions, iNeurons were co‐cultured with iMG (Figure 2d–f). As expected, numerous ePtdSer^+^ signals were detected while neurons were dying (Figure 2e). Interestingly, a significant number was colocalized with pHrodo‐oAβ signals, indicating the generation of ePtdSer^+^‐Aβs (white arrows; Figure 2e). Moreover, 4D live‐cell imaging and IMARIS rendering system revealed that ePtdSer^+^‐Aβs were engulfed by iMG (Figure 2f, Video S4, Supporting Information). Collectively, these results confirmed a possible regulation of microglial Aβ phagocytosis via PtdSer. Next, we investigated whether TREM2 mediates phagocytosis of ePtdSer^+^‐Aβs.

### 2.3 TREM2 Mediates Microglial Phagocytosis toward ePtdSer^+^‐Aβ

**Figure 3:**
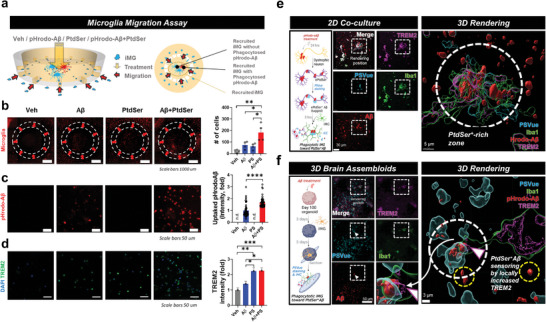
Microglia gravitate toward ePtdSer^+^ Aβs via TREM2. a) Schematic illustration of microglia migration chip. Four independent treatment conditions (‘No treatment’ vs ‘pHrodo‐oAβ’ vs ‘PtdSer’ vs ‘pHrodo‐oAβ + PtdSer’) were compared. b) Microglia gravitate toward ePtdSer^+^ Aβs. The number of cells was counted. c) Comparison of pHrodo‐oAβ intensity (uptaked pHrodo‐oAβ) between the ‘pHrodo‐oAβ’ and ‘pHrodo‐oAβ + PtdSer’ condition. d) Comparison of TREM2 intensity. e) Microglia gravitate toward Aβs in the PtdSer^+^ rich zone (2D co‐culture). 3D rendering image shows engulfment of ePtdSer^+^ Aβs by microglia via TREM2. Scale bar = 30 µm or 5 µm. f) Microglia gravitate toward ePtdSer^+^ Aβs (white circle) rather than Aβs alone (yellow circle) via TREM2 in the brain assembloid. 3D rendering image shows the captured moment of the microglial arm with high expression of TREM2 toward ePtdSer^+^ Aβs. For (a‐d), *p < 0.05, **p < 0.01, ****p < 0.0001, multiple comparisons (ANOVA) with Tukey's post‐hoc test or unpaired *t*‐test were used for statistical analyses. Microglia, pseudo‐colored; pHrodo‐oAβ, red; DAPI, blue; TREM2 green. For (e,f), Scale bar = 50 µm or 3 µm. White box, 3D rendering position; white circles for (e), PtdSer^+^ rich zone; white circle for (f), ePtdSer^+^ Aβ sensing via TREM2; yellow circle for (f), Aβ alone; pink arrow, the TREM2‐expressing microglial arm for sensing ePtdSer^+^ Aβ; blue, PSVue; green, anti‐Iba1; red, MX04; pink, anti‐TREM2.

To investigate whether TREM2 mediates phagocytosis of ePtdSer^+^‐Aβs, iMG migration assay, 2D co‐culture (iNeurons+iMG), 3D brain assembloids (3D brain organoids with iMG), and the brains of *APP^NL‐G‐F^*/*MAPT* dKI mice were cryo‐sectioned and visualized (**Figure** **3**). First, we performed iMG migration assay using chemotactic chip (Figure 3a). After adding culture medium supplemented with PtdSer (1 µg mL−1), pHrodo‐oligomeric Aβ (pHrodo‐oAβ) (2 µM), or PtdSer+ pHrodo‐oAβ to the central chamber, we compared the number of recruited iMGs, phagocytosed pHrodo‐ oAβs, and TREM2 intensity between each condition. Interestingly, the number of recruited iMG was significantly increased in PtdSer+ pHrodo‐oAβ condition, compared to vehicle, PtdSer, or pHrodo‐oAβ treatments (Figure 3b). We also validated that PtdSer can increase the phagocytic ability of iMG (Figure 3c) and confirmed that the level of TREM2 is significantly increased in the same condition (Figure 3d). It means that PtdSer can be an attractor for iMG toward beta‐amyloid peptides. Moreover, In the 2D co‐culture model, iMG gathered into a PtdSer‐rich zone and exhibited higher expression levels of TREM2 (white box; Figure 3e). Three‐dimensional rendering imaging revealed magnified ePtdSer^+^‐Aβs and the surrounding bunch of TREM2^+^ Iba1 signals (white circle; Figure 3e). We also captured a phagocytosing microglial arm stretching out toward ePtdSer^+^‐Aβ in a brain assembloid (white box; Figure 3f). Notably, the leading edge of the microglial arm showed a partial increase in TREM2, indicating that microglia sense ePtdSer^+^‐Aβs via TREM2 (white circle; Figure 3f). We also confirmed a significant increase in the levels of TREM2 due to the treatment of PtdSer by western blot analysis (Figure S6a, Supporting Information). Unfortunately, we could not get significantly different TREM2 levels by the bulk RNA seq (P > 0.05). However, we could confirm that the Top DEGs (MMP1, IGFBP3, IGF2R, IL1A) are interacting with TREM2 by using the STRING protein‐protein interaction (PPI) database (https://string‐db.org/) (Figure S6b–g, Supporting Information).

**Figure 4:**
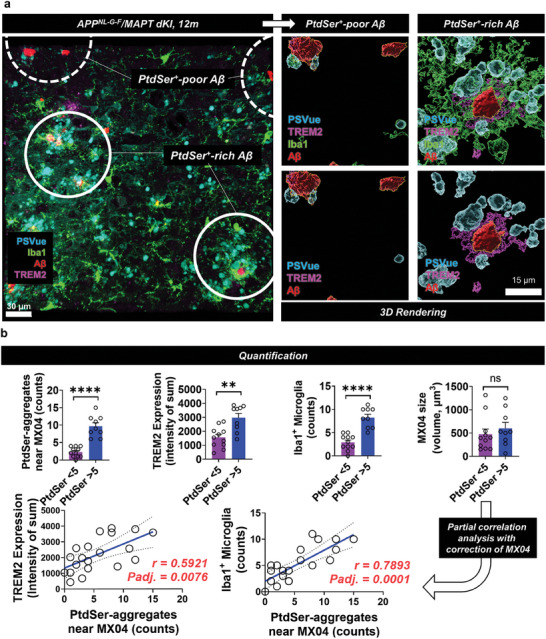
Difference in microglial responses toward Aβ plaques between PtdSer^+^ rich and poor zones in *APP^NL‐G‐F^*/*MAPT* dKI mice (12 months). a) The active microglia (high expression of TREM2) and the number of microglia (Iba1^+^) were significantly increased in the PtdSer^+^ rich zone. b) Quantification and correlation data. *p < 0.05, ***p < 0.001, independent *t*‐test; adjusted *p*‐values, *p*‐values from partial correlation analysis with the correction of MX04 (Aβ plaques) size; white circles for (a), PtdSer^+^ rich zone or PtdSer^+^ poor zone; blue, PSVue; green, anti‐Iba1; red, MX04; pink, anti‐TREM2. In N = 4 slices, MX04 regions (n = 12 for PtdSer aggregates < 5 and n = 8 for PtdSer aggregates> 5; total n = 20 spots; PtdSer aggregates were counted by IMARIS software after 3D rendering) were selected and quantified.

Next, since it is necessary to examine how many ePtdSer^+^‐Aβs exist and their relevance for TREM2^+^ microglia in the brain of the AD mouse model, brain cortex tissues from *APP^NL‐G‐F^*/*MAPT* dKI mice were cryosectioned and visualized (**Figure** **4**; Figure S7, Supporting Information). Although a different AD mouse model was used, a large portion of Aβ plaques were surrounded by ePtdSer, as reported previously by Huang *et al.* (Figure 4a; Figure S7a, Supporting Information).^[^ ^7^ ^]^ Consistent with the co‐culture and 3D brain assembloid models, the greater number of microglia gathered in the PtdSer‐rich zone (PtdSer > 5) and showed significantly higher expression levels of TREM2 (lower two white circles; Figure 4a), although the size of the Aβ plaques did not differ significantly (quantification data; Figure 4b). Moreover, the PSVue signal intensity was significantly correlated with the expression levels of TREM2 and microglial (Iba1) counts (Figure 4b). It should be noted that microglia did not gather in the PtdSer‐poor Aβ plaques (upper two white circles of Figure 4a; Figure S7b, Supporting Information). Thus, we believe that TREM2 may mediate the microglial phagocytosis of ePtdSer^+^‐Aβs.

### 2.4 Loss‐of‐Function of TREM2 Leads to Deficient Microglial Uptake of ePtdSer^+^‐Aβ

**Figure 5:**
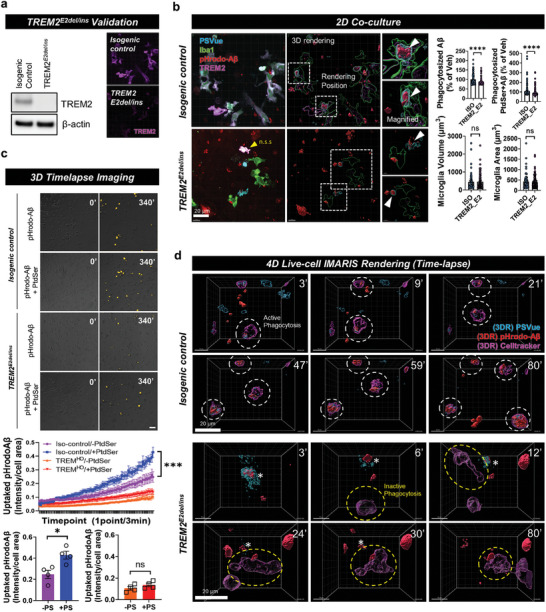
Loss‐of‐function of TREM2 causes defects in ePtdSer^+^‐Aβ recognition and phagocytic activity. a) Validation of iPSC‐derived microglia with TREM2 E2^del/ins^ mutation. b) Deficient uptake efficiency of TREM2 E2^del/ins^ microglia toward ePtdSer^+^ Aβ. n.s.s. non‐specific signals; white boxes, 3D rendering position. Scale bar = 20 µm. c) Timelapse imaging for TREM2 isogenic and TREM2 E2^del/ins^ microglial uptake of pHrodo‐Aβs with/without PtdSer. PtdSer treatment significantly increased the phagocytic activity of isogenic microglia but not TREM2 E2^del/ins^ microglia. Yellow, pHrodo‐Aβ. N = 4 slides for each group; average n = 129.5 cells were tracked per one field of view (scale bar = 100 µm). d) 4D time‐lapse imaging with 3D rendering using IMARIS software to compare between TREM2 isogenic and TREM2 E2^del/ins^ microglia. TREM2 E2^del/ins^ microglia show deficient uptake of ePtdSer^+^ Aβ. White circle, phagocytotic TREM2 isogenic microglia; yellow circle, deficient phagocytic activity of TREM2 E2^del/ins^ microglia; blue, PSVue; red, pHrodo‐Aβs; pink, cell‐tracker 647, microglia. Scale bar = 10 µm. ***p < 0.001, comparison of slope analysis for linear regression models; *p < 0.05, independent *t*‐test; ns, no significance.

To validate whether loss of TREM2 leads to defects in microglial phagocytosis of ePtdSer^+^‐Aβs, TREM2 mutant iMG (loss‐of‐function through a frameshift mutation in TREM2 exon 2 in both alleles; homozygous; P59AfsTer16 and P59AfsTer46) and isogenic normal control iMG were used.^[^ ^14^ ^]^ (**Figure** **5**a). In contrast to normal isogenic iMG phagocytosing ePtdSer^+^‐Aβs well, TREM2 mutant iMG showed deficient phagocytic activity toward ePtdSer^+^‐Aβs (Figure 5b; white arrows). However, morphological differences in microglia relevant to activation status were not observed (Figure 5b), indicating that other genes involved in microglial activation may act as a compensatory backup for maintaining their status (e.g., *LPL*, *CD33*, *CX3CR1*, etc.; Figure S8, Supporting Information). Moreover, to identify whether deficient microglial uptake of ePtdSer^+^‐Aβ was a TREM2‐dependent behavior, 3D long‐term timelapse imaging was conducted (Figure 5c). Interestingly, normal TREM2‐isogenic iMG showed a significant increase in phagocytic capacity toward Aβs after treatment with PtdSer, whereas TREM2 mutant iMG did not (Figure 5c), indicating that microglial uptake of ePtdSer^+^‐Aβ depends on the function of TREM2. Finally, 4D live‐cell imaging with 3D rendering tracking confirmed that TREM2 mutant iMG showed less active phagocytosis toward ePtdSer^+^‐Aβs and passed by Aβs without engulfment (Figure 5d, Video S6, Supporting Information), whereas normal TREM2‐isogenic iMG showed active phagocytosis toward ePtdSer^+^‐Aβs (Figure 5d, Video S5, Supporting Information). Our hypothesis that microglia gravitate toward ePtdSer^+^‐Aβs via TREM2 was thus confirmed.

### 2.5 AD Disease Models Showed Reduced Levels of TREM2 and a Lack of Phagocytic Activity toward ePtdSer^+^‐Aβs

**Figure 6:**
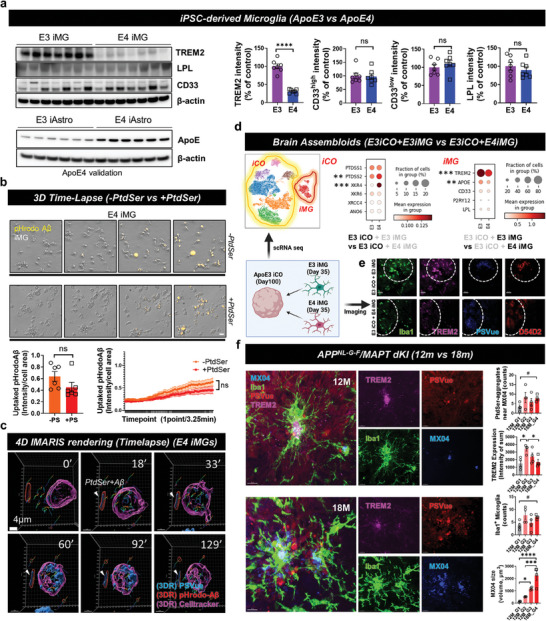
Loss‐of‐function of TREM2 toward ePtdSer^+^‐Aβ in both sporadic and familial AD model. a) Comparison of microglial markers between ApoE ɛ4/ɛ4 and ɛ3/ɛ3 iMG (upper band set) and validation of ApoE genotypes with iPSC‐derived astrocytes (iAsts; lower band set). Due to the low expression levels of ApoE proteins in iMG, we used iAsts for *APOE* genotype validation. ^****^ *p* < 0.0001, independent *t*‐test. b) The 3D long‐term live‐cell imaging shows that PtdSer treatment does not affect the phagocytic activity of ApoE ɛ4/ɛ4 iMG. N = 6 slides for each group; average n = 30.38 cells were tracked per one field of view (scale bar = 20 µm). *P*‐values by independent *t‐*test and comparison of area under curves. c) 4D time‐lapse imaging with 3D rendering using IMARIS software. ApoE ɛ4/ɛ4 iMG show deficient phagocytic activity on ePtdSer^+^ Aβ. blue, PSVue; red, pHrodo‐Aβs; pink, cell‐tracker 647, microglia. Scale bar = 4 µm. d) Comparison of ApoE ɛ4/ɛ4 and ɛ3/ɛ3 iMGs with single cell RNA sequencing using human brain assembloids. Dot plots show the expression levels and fraction of cells that express each gene in iCOs and iMG. Statistical significance of differentially expressed genes and *P*‐values were calculated using Wilcoxon rank sum test for each group. e) Comparison of ePtdSer^+^ Aβs and microglial TREM2 in brain assembloids between ApoE ɛ4/ɛ4 and ɛ3/ɛ3 brain assembloids. blue, PSVue; green, anti‐Iba1; red, Aβ, anti‐D54D2; pink, anti‐TREM2. Scale bar = 50 µm. f) Comparison of ePtdSer^+^ Aβs and microglial TREM2 in the brain cortical sections of APP/MAPT dKI mice between 12‐month‐old mice and 18‐month‐old mice. 12 month‐group 1 (12M_G1), MX04 volume < 500; 12 month‐group 2 (12M_G2), 500 < MX04 volume < 1000; 12 month‐group 3 (12M_G3), MX04 volume > 1000; 18 month‐group 4 (18M_G4), MX volume > 1000. Sequential groups represent the progression of AD (from acute to chronic status). N = 4 slices were used for each bar. blue, PSVue; red, anti‐Iba1; blue, Aβ (MX04); pink, anti‐TREM2. Scale bar = 10 µm. ^#^p = 0.05, independent *t*‐test; *p < 0.05, ***p < 0.001, and ****p < 0.0001 by ANOVA with post‐hoc test; ns, no significance.

Since many studies have shown loss‐of‐function or decrease in TREM2 expression in AD,^[^ ^15^ ^]^ both sporadic (CRISPR‐Cas9‐based *APOE4* lines; validation with iPSC‐derived astrocytes in **Figure** **6**a) and familial (*APP^NL‐G‐F^*/*MAPT* dKI mice) AD models were used for validating TREM2‐dependent defect in microglial phagocytosis. As expected, the levels of microglial TREM2 protein and mRNA in *APOE4* iMG were decreased, whereas no changes were observed in the levels of other proteins or mRNAs related to microglial function (Figure 6a; Figure S8, Supporting Information). In contrast to the increase in phagocytic activity in PtdSer‐treated *APOE3* iMG (Figure 2a,b, Video S2, Supporting Information), *APOE4* iMG did not show increased phagocytic activity upon PtdSer treatment (Figure 6b, Video S7, Supporting Information). Our 4D live‐cell imaging with 3D rendering tracking also showed a lack of phagocytic activity of *APOE4* iMG toward ePtdSer^+^‐Aβ (Figure 6c, Video S8, Supporting Information). These results indicate that the phagocytosis of *APOE4* iMG cannot be modulated through PtdSer treatment, possibly due to a lack of functional TREM2. Next, to compare *APOE3* iMG and *APOE4* iMG in an advanced 3D co‐culture system, the iMG were co‐cultured with Aβ‐treated *APOE3* iCOs (Figure 6d,e; *APOE3* iCO + *APOE3* iMG vs *APOE3* iCO + *APOE4* iMG), and then, each “brain assembloid” was used for scRNA‐seq or immunohistochemistry. Interestingly, scRNA‐seq data revealed higher expression of PtdSer‐related genes (e.g., *PTDSS, XKR4*) in *APOE4* iMG‐mixed *APOE3* iCOs (Figure 6d) than in *APOE3* iMG‐mixed *APOE3* iCOs, indicating that iCOs present more PtdSer signals when mixed with *APOE4* iMG rather than *APOE3* iMG. We also confirmed that the levels of *TREM2 and APOE* mRNA expression were significantly decreased in *APOE4* iMG (Figure 6d) whereas no changes for *CD33 and P2RY12* mRNA expression were observed in *APOE4* iMG. This phenomenon was also confirmed via immunohistochemistry image analysis. Consistent with the results of scRNA‐seq analysis, *APOE4* iMG showed a notable decrease in TREM2 expression, and *APOE4* iMG‐mixed iCOs showed more Aβ plaques (D54D2) and PtdSer (PSVue) signals than did *APOE3* iMG‐mixed iCOs (Figure 6e). Moreover, we tried to mimic AD disease progression with 12‐months‐old and 18‐months‐old *APP^NL‐G‐F^*/*MAPT* dKI mice by their plaque sizes (MX04 volume) (12 month‐group 1 (12M_G1), MX04 volume < 500; 12 month‐group 2 (12M_G2), 500 < MX04 volume < 1000; 12 month‐group 3 (12M_G3), MX04 volume > 1000; 18 month‐group 4 (18M_G4), MX volume > 1000) (Figure 6f). Interestingly, 18M_G4 mice (much more severe model than 12M_G1 to G3) showed reduced levels of TREM2 near the Aβ plaques than did 12M_G2 mice despite higher levels of Aβ plaques (Methoxy‐X04), and 12M_G2 mice showed increased levels of TREM2 near the Aβ plaques than did 12M_G1 mice (Figure 6f). This indicates that TREM2 levels can rise during acute conditions (12M), but subsequently decline during chronic conditions (18M), and finally, microglial cells lose their phagocytic activity in the chronic disease situation. Thus, we concluded that loss‐of‐function of TREM2 is observed in both sporadic and familial AD models.

## 3 Discussion

In this study, we focused on TREM2‐dependent microglial behavior toward Aβs in the context of the presence of ePtdSer. We considered the fact that there have not been many reports about what factors can attract microglial cells to pathological proteins such as Aβ plaques or neurofibrillary tangles, although TREM2 has gained increasing attention due to its significant role in microglial phagocytosis.^[^ ^16^ ^]^ To assess this phenomenon from a different standpoint, we took our cue from the fact that normal microglial cells can engulf the dystrophic synapses displaying ePtdSer as an “eat‐me” signal to maintain a normal healthy brain through synaptic elimination.^[^ ^5b^ ^]^ Thereafter, we thought of a what‐if scenario that synapses are highly affected by extracellular Aβs and are externalizing too many ePtdSers to be cleared during the progression of neurodegenerative disease such as AD, not in the normal status of the brain.

We speculated that many aggregates of ePtdSer produced by dystrophic neurons may become entangled with Aβ plaques during the progression of AD. Interestingly, we found a study, with results that were consistent with our speculation, by Huang *et al.*, they observed that ePtdSer surrounded Aβ plaques in the brains of *APP/PS1* mice and mentioned that ePtdSer‐associated Aβ plaques (ePtdSer^+^‐Aβs) may indicate the dystrophic neurites from damaged neurons.^[^ ^7^ ^]^ We therefore validated this phenomenon by ourselves using co‐culture systems and animal models and observed that ePtdSers were externalized from dystrophic neurons and were highly colocalized with Aβs (ePtdSer^+^‐Aβs) in an AD co‐culture model, 3D brain assembloids, and the brains of *APP^NL‐G‐F^*/*MAPT* dKI mice (Figures 2, 3, 4, 5, 6). Additionally, we thought that it was necessary to identify whether PtdSer is an initiation factor for the phagocytosis of Aβ plaques. Through long‐term live cell imaging with iMG, PtdSer, and pHrodo‐oAβs, we proved that PtdSer is one of the initiators or boosters of Aβ phagocytosis (Figure 2a–c, Videos [Link], [Link], Supporting Information). Moreover, short‐term 3D live cell confocal imaging with our co‐culture system including iNeurons and iMG showed that iMG engulfed ePtdSer^+^‐Aβs which were generated from dystrophic iNeurons (Figure 2f, Video S4, Supporting Information).

Next, after confirming that PtdSer can attract microglial cells toward Aβs, we investigated whether microglial engulfment of ePtdSer^+^‐Aβs can be mediated by TREM2 since TREM2‐PtdSer mediated synaptic engulfment was suggested by other previous reports.^[^ ^4^, ^5^ ^]^ First, we captured the moment that microglial cells elongate their arms and enrich the expression of TREM2 at the contacting part of the arm toward ePtdSer^+^‐Aβs in a 3D brain assembloid (Figure 3b), indicating that microglia sense ePtdSer^+^‐Aβs via TREM2. In addition, the immunohistochemistry for brains of *APP^NL‐G‐F^*/*MAPT* dKI mice showed a correlation between the expression of TREM2 and ePtdSer^+^‐Aβs (Figure 4), and loss‐of‐function of TREM2 iMG also revealed that microglial engulfment of ePtdSer^+^‐Aβs is mediated by TREM2 (Figure 5, Videos [Link], [Link], Supporting Information). Also, although there were no significant changes in the migration activity with respect to the treatment of PtdSer, an increasing tendency after the treatment of PtdSer in the TREM2 isogenic iMGs was shown in Figure S9d (Supporting Information) (p = 0.09). We confirmed that TREM2 loss‐of‐function could not induce migration activity. For APOE iMG lines, Both APOE3 and APOE4 microglia showed significantly increased migration activity after the treatment of PtdSer (Figure S9e, Supporting Information). Of course, the increasing tendency had much lower significance in APOE4 than in APOE3, we speculate that the reason is that APOE4 showed much lower expression levels of TREM2. Therefore, from Figure S9 (Supporting Information), we have shown again that the loss‐of‐function TREM2 means their loss of responses to PtdSer. In sum, all these data led us to believe that microglial cells gravitate toward Aβs surrounded by ePtdSer via TREM2.

This study makes several noteworthy points as follows: i) it is unique wherein it focused on the role of PtdSer in Aβ phagocytosis by microglia rather than in normal synapse pruning or elimination; ii) it revealed the driving force behind TREM2‐mediated phagocytosis of beta‐amyloid (Aβ); iii) it used advanced iPSC‐based co‐culture models (2D iNeuron and iMG co‐culture, 3D brain assembloids including brain organoids and iMG, etc.) and even an animal AD model (*APP^NL‐G‐F^*/*MAPT* dKI mice) to address TREM2‐dependent microglial phagocytosis; iv) during the study, microglial phagocytosis was visualized with a variety of unique and high‐end imaging techniques such as long‐term 2D live cell imaging with Image Exfluorer,^17^ ^]^ short‐term 3D live cell imaging with multi‐pinhole‐based spinning microdisk confocal microscope,^[^ ^18^ ^]^ and 3D/4D IMARIS rendering system.^[^ ^9^, ^19^ ^]^ However, of course, there were some limitations to this study: i) we did not address the exact binding site of TREM2, and whether TREM2 binds to PtdSer or Aβ in ePtdSer^+^‐Aβs remains unknown; ii) it is necessary to perform live cell imaging with brain assembloids using tissue‐based imaging techniques such as multi‐photon microscopy; iii) further validations using TREM2 mutant mouse models are required. Despite these limitations, we believe that this paper provides a new insight into microglial phagocytosis of Aβ via the TREM2‐PtdSer axis.

## 4 Experimental Section

### Human Induced Pluripotent Stem Cell (hiPSC) Culture

hiPSCs were maintained as described previously.^[^ ^11^ ^]^ Briefly, hiPSCs were detached using ReLeSR (ST05872, Stemcell Technologies) in 6‐well plates (30006, SPL) coated with Matrigel hESC‐qualified Matrix (354277, Corning) and maintained using mTeSR plus medium (ST05825, Stemcell Technologies) with Y‐27632 (ST72304, Stemcell Technologies). The next day, the medium was replaced with mTeSR plus medium lacking Y‐27632 and incubated at 37 °C in 5% CO_2_. Thereafter, the medium was replaced with a fresh medium every two days, and cells were passaged every 5 days using ReLeSR.

### Generation of hiPSC‐Derived Microglia (iMG)

iMG were generated as described previously.^[^ ^20^ ^]^ Briefly, hematopoietic progenitor cells (HPCs) were generated using STEMdiff Hematopoietic kit (ST05310, Stemcell Technologies). On day ‐1, hiPSCs were cultured in mTeSR plus medium (ST05825, Stemcell Technologies) and maintained using ReLeSR (ST05872, Stemcell Technologies) and Matrigel hESC‐qualified Matrix (354277, Corning) coated plates with mTeSR plus medium. On day 0, when 20–40 surviving clusters were generated, the culture medium was replaced with STEMdiff Hematopoietic kit medium A. On day 2, 500 ul of medium A was added to each well, and the cells were incubated at 5% CO_2_ and 37 °C. On day 3, previous media were removed, and then medium B was added. On days 5, 7, and 10, 500 ul of medium B was added to each well again. On day 12, the HPCs were transferred to a 15‐ml conical tube and centrifuged at 300 × *g* for 5 min. After centrifugation, HPCs were collected and plated at a density of 200,000–250,000/well in a 6‐well plate coated with Matrigel Growth Factor Reduced Basement Membrane Matrix (354230, Corning) and maintained in iMG differentiation medium containing DMEM/F12 (11039021, Gibco), 2% insulin‐Transferrin‐Selenium (11039021, Gibco), 1% MEM Non‐Essential Amino Acids Solution (11140050, Gibco), 2% B‐27 Supplement, serum‐free (17504‐044, Gibco), 0.5% N‐2 Supplement (17502‐048, Gibco), 1% GlutaMAX (35050‐061, Gibco), 400 uM 1–Thioglycerol (M1753‐100ML, Sigma), and 5 ug ml^−1^ Human insulin (I2643‐25MG, Sigma). Next, 1 mL iMG‐differentiation medium with tri‐cytokine cocktails (100 ng/ml IL‐34 (200‐34, Peprotech), 50 ng/mL TGFβ‐1 (100‐21 Peprotech), and 25 ng mL^−1^ M‐CSF (300‐25 Peprotech)) were added every other day from day 1 to day 25. On day 12, cells were centrifuged again at 300 × *g* for 5 min and resuspended in 2 mL of fresh iMG‐differentiation medium with tri‐cytokine cocktails. On day 25, cells were centrifuged at 300 × *g* for 5 min and resuspended in 2 mL fresh iMG differentiation medium with five‐cytokine cocktails (100 ng/mL CD200 (Bon Opus Bio, BP004), 100 ng/mL CX3CL1 (300‐31, Peprotech), 100 ng mL^−1^ IL‐34, 50 ng mL^−1^ TGFβ1, 25 ng mL^−1^ M‐CSF). The iMG differentiation medium with five‐cytokine cocktails was added every other day from day 25 to day 37. For TREM2 loss‐of‐function experiments, a commercialized iMG line purchased from Fujifilm (iCell Microglia AD TREM2, R1201; iCell isogenic control, R1131) was used.

### Generation of hiPSC‐Derived Brain Organoids (iCOs)

iCOs were generated as described previously.^[^ ^11^ ^]^ Briefly, hiPSCs were detached with ReLeSR, dissociated into single cells, and then loaded into AggreWell800 24‐well plates (34811, Stemcell Technologies) with EB formation medium (ST05893, Stemcell Technologies) containing the ROCK inhibitor, Y‐27632, to form embryoid bodies (EB). The next day, the medium was replaced with an EB formation medium lacking Y‐27632. From day 2–5, the medium was replaced daily with DMEM/F‐12 containing GlutaMAX (10565‐018, Gibco), 20% KnockOut Serum Replacement (A3181501, Gibco), and 1% MEM non‐essential amino acid solution (11140050, Gibco), 0.1 mM 2‐mercaptoethanol (21985023, Gibco), 100 U/ml penicillin, and 100 µg ml^−1^ streptomycin (P4333, Merck), dorsomorphin (10 µM; P5499SB‐Merck), and SB‐431542 (10 µM; 1614, TOCRIS). The EBs were collected on day 6 and seeded the next day in each well of ultra‐low attachment 96‐well plates (7007, Corning). iCOs were cultured with Neurobasal‐A medium (10888‐022, Gibco), B‐27 supplement minus vitamin A (12587010, Gibco), 100 U/ml penicillin, 100 µg ml^−1^ streptomycin, GlutaMAX (35050‐061, Gibco), and 0.5% (v/v) Matrigel Basement Membrane Matrix (354234, Corning). From day 7 to day 24, the medium was replaced daily with neurobasal medium supplemented with 20 ng ml^−1^ epidermal growth factor (EGF; 01–107, Merck) and 20 ng ml^−1^ fibroblast growth factor basic (bFGF; 233‐FB, R&D Systems). From day 25 to day 42, the medium was changed to a neural medium supplemented with 20 ng ml^−1^ brain‐derived neurotrophic factor (BDNF; 450‐02, Peprotech) and 20 ng/ml neurotrophin‐3 (NT‐3; 450‐03, Peprotech) every other day. From day 42 to day 100, the medium was replaced with a neurobasal medium without Matrigel basement Membrane Matrix and growth factors every 4 days.

### Generation of Brain Assembloids

The iCOs were carefully seeded into individual wells of an ultra‐low attachment 24‐well plate (3473, Corning) using universal wide bore bulk tips (T521017, TARSONS). Next, iMGs (200,000 cells) were added to each well where a single brain organoid was cultured. According to the previous report, iCOs and iMGs were co‐cultured in a neurobasal medium with five cytokines (IL‐34, TGFβ1, M‐CSF, CD200, and CX3CL1).^[^ ^21^ ^]^ Brain assembloids were incubated on a shaker for 3–7 days, used for immunohistochemistry, and analyzed with IMARIS software (Bitplane, Zurich, Switzerland).^[^ ^19^, ^22^ ^]^

### Migration Chip Fabrication

Details of the migration chip design were described in the previous study.^[^ ^23^ ^]^ To fabricate a mold of the device, a SU‐8 negative photoresist (MicroChem, Round Rock, TX), was sequentially patterned using photolithography on a silicon wafer. A mixture of base and curing agent of Sylgard 184 A/B polydimethyl‐siloxane (PDMS) (Dow Corning, Midland, MI) was poured onto the SU‐8 mold to replicate the microstructures. The cured PDMS replica was removed from the mold, and holes were created for fluid reservoirs. Plastic chambers for media reservoirs were fabricated with a computer‐controlled Zing laser cutter (Epilog Laser, Golden, CO) with a 6‐mm‐thick acrylic plate. The replicated PDMS and plastic layers were glued together using PDMS. The resultant assembly was irreversibly bonded to a customized glass‐bottomed uni‐well plate (MatTek, Ashland, MA) by oxygen plasma treatment (Plasma Etch, Carson City, NV).

### iMG Migration Assay

The chemotactic chip was employed for the migration assay. Before the migration assay, each chamber was coated with 1% (v/v) Matrigel matrix (Corning) diluted in DMEM/F‐12 for 1 hr and washed it with phosphate‐buffered saline (PBS) thoroughly. Afterward, 1×104 iPSC‐derived microglia was loaded in the annular chamber while culture medium was added supplemented with PtdSer (1 µg mL−1), pHrodo‐oligomeric Aβ (pHrodo‐oAβ) (2 µM), or PtdSer+ pHrodo‐oAβ to the central chamber. After 2 days of incubation in a 5% CO2 cell culture incubator at 37°C, the number of microglia migrating to the central chamber was counted by using a fully automated fluorescence microscope (Nikon Ti2E microscope, Nikon, Melville, NY).

### Immunocytochemistry for Migration Chip

Upon the completion of microglia migration, the chemotactic chips were rinsed with PBS twice and fixed with 4% paraformaldehyde (PFA, Electron Microscopy Sciences, Hatfield, PA) for 30 min at RT. The models were then rinsed with PBS two times at 10 min intervals and incubated in the permeabilizing solution, PBS solution supplemented with 0.1% (v/v) Triton X‐100 and 0.1% (v/v) Tween 20 (PBST), for 30 min at RT. Cells were next washed with PBS three times at 10‐minute intervals and incubated in the blocking solution, PBS solution supplemented with 0.1% (v/v) Tween 20 and 3% (v/v) human serum albumin (BSA), for 2 hrs at RT. Cells were again washed with PBS three times at 10‐minute intervals and incubated with the primary antibody diluted in the blocking solution in a 1:100 ratio (v/v). After the secondary antibody reaction, the devices were washed seven times with PBS supplemented with 0.1% (v/v) Tween 20 (PBST) at 10‐minute intervals and examined under a fluorescence microscope (Nikon Ti2‐E microscope, Nikon). It should be noted that the central chamber was focused on in which only activated microglia were presented. The intensity of immunoreactivity was analyzed by using NIS‐Elements software.

### Single‐Cell RNA Sequencing (scRNA‐seq) Using Brain Assembloids

Brain assembloids were dissociated using an adult brain dissociation kit (Miltenyi Biotec) with MACS octo dissociator with heater (Miltenyi Biotec) using 37C_ABCK_2. Dissociated cells were filtered through a 70‐um SmartStrainer (Miltenyi Biotec) to produce separated single cells. For live cell sorting, Fixable viability stain 700 (BD) was used to label dead cells which were filtered out using SH800S FACS (Sony). Sorted live cell suspension was centrifuged at 300 × *g* (4 °C) for 5 min and then suspended in 0.04% BSA solution. Then, 10×3’ gene expression libraries were generated using Chromium Next GEM Chip G and Chromium Next GEM Single Cell 3’ Kit v3.1 (10x Genomics).^[^ ^24^ ^]^ All libraries were sequenced using the NovaSeq 6000 system (Illumina) to generate paired‐end 100bp.

### Single‐Cell RNA Sequencing (scRNA‐Seq) Analysis

All sequenced data were aligned to the GRCh38 reference genome using Cell Ranger (v6.0.1).^[^ ^25^ ^]^ Only cells expressing more than 200 genes, not expressing more than 1% mitochondrial genes were used for further analysis. Doublets were removed Scrublet package (v0.2.3).^[^ ^26^ ^]^ Next, expression level normalization was conducted using CPM normalization in the SCANPY package (v1.9.6) regressing out a percentage of mitochondrial genes.^[^ ^27^ ^]^ For batch correction, the SCVI package (v0.6.8) was used to integrate libraries.^[^ ^28^ ^]^ The statistical significance of differentially expressed genes was calculated using the Wilcoxon rank sum test for each group.

### Generation of hiPSC‐Derived Neural Progenitor Cells (NPCs)

NPCs were generated as described previously.^[^ ^29^ ^]^ hiPSCs were transduced with lentiviruses encoding TetO‐Ngn2‐GFP‐Puro and reverse tetracycline‐controlled transactivator (rtTA) and were plated in a 6‐well plate coated with Geltrex LDEV‐Free hESC‐qualified Reduced Growth Factor Basement Membrane Matrix (A1413302, Thermo) at a density of 75,000 cells per cm^2^ in mTeSR Plus medium containing Y‐27632. On day 0, the medium was replaced with DMEM:F12 + Glutamax (10565018, Gibco) containing 1.5% v/v 20% Glucose (A2494001, Gibco), 1% N2 Supplement (17502‐048, Gibco), SB431542 (10 µM;1614,TOCRIS), XAV939 (2 µM; X3004‐25MG, Sigma), LDN‐193189 (200 nM; 6053, TOCRIS), and doxycycline hyclate (2 µg ml^−1^; D9891‐1G, Sigma). On Day 1, the medium was replaced with DMEM/F12 + Glutamax, 1.5% v/v 20% Glucose, 1% N2 Supplement + 5 µM SB, 1 µM XAV, 100 nM LDN with puromycin (5 µg mL^−1^; P8833 Sigma). On day 2, NPCs were dissociated into single cells using Accutase (A1110501, Gibco) with Y‐27632, seeded at a density of 125,000 cells cm^−2^ on Geltrex‐coated plates, and incubated in a 3 mL/well NPC maintenance medium (DMEM/F12 + Glutamax, 1% Glutamax, 100 U/ml penicillin and 100 µg ml^−1^ streptomycin, 1% MEM non‐essential amino acid solution, B‐27 without Vitamin A (12587010, Gibco)), N‐2 Supplement (17502‐048, Gibco), 10 ng ml^−1^ epidermal growth factor (EGF), 10 ng ml^−1^ fibroblast growth factor basic (bFGF), Puromycin, and Y‐27632. On day 3, the medium was replaced with NPCs maintenance medium lacking Puromycin and Y‐27632. Thereafter, the NPCs were passaged every 5–6 days on average.

### Generation of iPSC‐Derived Neurons (iNeurons)

iNeurons were generated as described previously.^[^ ^30^ ^]^ Briefly, NPCs were dissociated into single cells, were replated on Matrigel hESC‐qualified Matrix coated confocal dishes (100350, SPL), and maintained in a neural differentiation medium (Neurobasal‐A media, B‐27 Supplement minus vitamin A, 100 U ml^−1^ penicillin, and 100 µg ml^−1^ streptomycin), supplemented with 10 ng mL^−1^ BDNF, and 10 ng mL^−1^ NT‐3 for 14 days.

### Generation of iPSC‐Derived Astrocytes (iAstrocytes)

iAstrocytes were generated as described previously.^[^ ^31^ ^]^ Briefly, NPCs were detached using Accutase at 5% CO_2_ and 37 °C for 10 min and transferred to a 15‐ml conical tube. After astrocyte medium (1801, astrocyte medium, 2% fetal bovine serum, astrocyte growth supplement, antibiotic solution, ScienCell) was added in a 1:2 ratio, the cells were centrifuged at 300 × g for 5 min. Next, the cells were resuspended in 1 mL of astrocyte medium and dissociated into single cells. The cells (144,000 cells) were seeded on a Geltrex‐coated plate and maintained in an astrocyte medium containing Y‐27632. The next day, the medium was replaced with an astrocyte medium lacking Y‐27632. Thereafter, the medium was changed every 2 days, and the cells were passaged on average every 5 days. As described by Tcw et al., cells were cultured for more than 30 days to mature astrocytes.^[^ ^31^ ^]^ The generated astrocytes were used for *APOE* genotype validation through western blotting.

### Co‐culture of Human NPCs‐Derived Neurons and hiPSC‐Derived Microglia

iNeurons were differentiated from NPCs, plated on confocal plates at 10,000 cells per well on Matrigel hESC‐qualified Matrix‐coated plates, and matured for 14 days in a neural differentiation medium. iMG were plated on top of neurons at 200,000 cells per well in an iMG‐differentiation medium with two‐cytokines cocktails (100 ng ml^−1^ IL‐34, 25 ng mL^−1^ M‐CSF). The next day, they were used for immunocytochemistry and live cell imaging.

### Immunocytochemistry

Cells were fixed with 4% paraformaldehyde (PFA) (P2031, Biosesang) for 20 min at room temperature, washed with PBS, and blocked with 0.3% Triton X‐100 (X100, Merck) in PBS containing 5% bovine serum albumin (BSA; 10857, Affymetrix) for 2 h at room temperature. Primary antibodies were diluted in 0.3% PBST containing 1% BSA and incubated with the fixed cells at 4°C overnight. The next day, cells were washed with PBS and incubated with secondary antibodies at 1:500 for 1 h at room temperature in the dark. After secondary staining, cells were washed with PBS and mounted on slides. Confocal images were acquired using a multi‐pinhole‐based Spinning Microdisk Confocal (Nikon). The following primary antibodies were used: CD68 (1:500; MCA1957GA, Bio‐Rad), iba1 (1:250; 234308, SYSY), MAP2 (1:500; ab5392, Abcam), TREM2 (1:500; ab86491, Abcam).

### Immunohistochemistry of Brain Assembloids and Mouse Brain

Brain assembloids were washed with PBS and placed in 4% PFA at 4 °C overnight. The next day, they were washed with PBS to remove PFA and put in 30% sucrose at 4 °C for 72 h, and transferred to a Biopsy cryomold (4565, SAKURA) and frozen in FSC 22 Compound (3801480, Leica). Brain assembloids were sectioned and mounted on slices. They were stained with PSVue (1:500 in PBS; P‐1002 or P‐1005, MTTI) for 10 min at room temperature, washed 3 times with PBS, and permeabilized using 0.3% PBST for 30 minutes at room temperature (RT). After 30 min, blocking was performed for 1 h at room temperature using 5% normal horse serum (S‐2000, Vector Laboratories). Primary antibodies were diluted in 5% normal horse serum and incubated at 4°C overnight. The next day, primary antibodies were removed and washed 3 times with 0.3% PBST. Secondary antibodies were diluted at a ratio of 1:500 in 5% BSA in 0.3% PBST and treated for 1 h at room temperature. Slices were washed 3 times with PBS and mounted on slides. Confocal images were acquired using Spinning Microdisk Confocal (Nikon).

Mice were sacrificed at 12 and 18 months of age and perfused with PBS. Brain samples from mice were incubated in 4% PFA overnight for fixation, followed by incubation in 30% sucrose for 3 days. They were sliced (30‐um thick) in a cryostat and mounted on slides. Next, they were stained with PSVue (1:500 in PBS; P‐1002 or P‐1005, MTTI) for 30 minutes at room temperature, washed 3 times with PBS, and permeabilized using 0.3% PBST for 30 minutes at room temperature. After 30 minutes, blocking was performed for 1 h at room temperature using 5% normal horse serum (S‐2000, Vector Laboratories). Primary antibodies were diluted in 5% normal horse serum and incubated with slices at 4°C overnight. The next day, primary antibodies were removed, and slices were washed 3 times with 0.3% PBST. Next, methoxy‐X04 (1:200; 4920/10 or 4920/50, R&D Systems) was added to the secondary antibody solution (1:500 in 5% BSA and 0.3% PBST) and incubated for 1 h at room temperature. After 1 h, slices were washed 3 times with PBS and mounted on slides. Confocal images were acquired using a Spinning Microdisk Confocal Microscope (Nikon).

The following primary antibodies were used: GFAP (1:500; 130300, Invitrogen), IBA1 (1:250; 234308, SYSY), MAP2 (1:500; ab5392, Abcam), TREM2 (1:500; AF1828, R&D), TREM2 (1:500; ab86491, Abcam), β‐amyloid (D54D2) (1:300; 8243, Cell Signaling Technology).

### Reverse Transcriptase Quantitative PCR (RT‐qPCR)

Total RNA was isolated using RNeasy Plus Mini Kit (74136, QIAGEN) as per the manufacturer's instructions. cDNA was synthesized using Maxime RT PreMix Kit (25081, iNtRON Biotechnology). Real‐time quantitative PCR was performed using the SYBR Green Fast mix (KR0389, KABA biosystems). Data were analyzed using the StepOnePlus Real‐Time PCR System and the primers were listed in Figure S8 (Supporting Information). The Human 18s gene was used as a control for each sample as a housekeeping gene.

### Western Blotting

Cells were lysed with radioimmunoprecipitation assay (RIPA) buffer containing protease and phosphatase inhibitors (P‐8340 and 93482, Sigma Aldrich; P‐1517 and P‐1518, AG Scientific). Proteins were quantified using a bicinchoninic (BCA) protein assay. Following quantification, cell lysates were loaded into each well and separated on NuPAGE 4–12% Bis‐Tris gels (NP0323BOX, Thermo Fisher Scientific) in MES buffer (NP0002, Thermo Fisher Scientific) and then transferred to PVDF membranes (IPVH00010, Merck Millipore) for 90 min at 70 V. After transfer, the membrane was blocked with 5% skim milk in Tris‐buffered saline with 0.05% Tween 20 (TBST) for 1 h. After blocking, the membrane was incubated with primary antibodies (CD33, #77576, Cell Signaling Technology; LPL, ab21356, abcam; ApoE ε4, #39327, Cell Signaling Technology; TREM2, AF1828, R&D systems; beta‐actin, 3700s, Cell Signaling Technology) in TBST (with 3% BSA and 0.05% sodium azide) overnight at 4 °C. The next day, the membrane was washed 5 times with TBST and incubated for 1 h with secondary antibodies in 2.5% skim milk in TBST at RT. The membranes were imaged on a bio‐imaging analyzer (AI600, GE Healthcare Life Science, IL. USA) with a SuperSignal™ West Femto Maximum Sensitivity Substrate (34095, Thermo Fisher Scientific) and quantified with a Multi‐gauge 3.0 Software (Fujifilm Corporation, Tokyo, Japan).

### 2D Long‐Term Live‐Cell Imaging

For the pHrodo‐oAβ uptake assay, oligomeric Aβs (oAβs) were generated through incubation of monomeric Aβ at 4 °C for 18 h. After that, pHrodo iFL Red Microscale Protein Labeling Kit (P36014, ThermoFisher Scientific) was used for tagging pHrodo to oAβs. iMG were collected and plated at a density of 10,000/well of a 96‐well plate‐coated Matrigel Growth Factor Reduced Basement Membrane Matrix (354230, Corning) and maintained in iMG differentiation medium. After adding 2 uM pHrodo‐oAβ in iMG‐maintaining medium (iMG differentiation medium with 100 ng ml^−1^ IL‐34 and 25 ng ml^−1^ M‐CSF), the 96‐well plate was incubated at 37 °C, 5% CO_2_, for 30 min. After 30 min, just before recording live‐cell images, iMG‐maintaining media was treated containing 1 ug/ml PtdSer (840032P, Sigma) and PSVue (100X). The microglial uptake of pHrodo‐oAβs colocalized with PtdSer was imaged using the Image ExFluorer (Live Cell Instrument, Korea) system. The Video was recorded for 6 h and analyzed using ImageJ software (NIH Image).

For the bead uptake assay, iMG were collected and plated at a density of 100,000/well in a 12‐well plate‐coated Matrigel Growth Factor Reduced Basement Membrane Matrix (354230, Corning) and maintained in iMG‐differentiation medium. Beads were opsonized with 50% FBS after incubation in iMG maintaining medium (iMG‐differentiation medium with 100 ng ml^−1^ IL‐34 and 25 ng mL^−1^ M‐CSF) at 37 °C for 30 min. After 30 min, iMG were treated with opsonized beads for 1 h in the dark for the opsonization of beads. Then, the microglial uptake of the beads was recorded with the Image ExFluorer system. The Video was recorded for 6 h and analyzed using ImageJ software.

### 3D Short‐Term Live‐Cell Confocal Imaging

NPCs were dissociated into single cells using Accutase, seeded at 5,000 cells per well on Matrigel hESC‐qualified Matrix‐coated glass‐bottom dishes for confocal microscope imaging, and maintained for 14 days in a neural differentiation medium. After the maturation of neurons, neurons were treated with 2 µM pHrodo‐oAβ and incubated overnight at 37 °C and 5% CO_2_. The next day, iMG (200,000 cells) were labeled with CellTracker™ Deep Red Dye (C34565, Thermo Fisher Scientific) in iMG maintaining medium at 37 °C and 5% CO_2_ for 1 h. After staining, labeled iMG were washed with PBS. After labeling iNeurons with Memglow‐488 (MG01, cytoskeleton, Inc.) for 5 min, microglia were plated on top of the neurons and maintained in iMG‐maintaining medium with PSVue (100X). The co‐cultured cells were recorded with Spinning Microdisk Confocal for 2 h. 3D rendering for 3D/4D live‐cell imaging was performed using IMARIS Software (Oxford Instruments and Bitplane).

### Animals

All animal experiments were conducted according to the Animal Care and Use Guidelines of Seoul National University, Seoul, Korea (Institutional Animal Care and Use Committee; IACUC). *APP^NL‐G‐F^* and *MAPT* knock‐in mice carrying *APP^KM670/671NL^* (Swedish), *APP^I716F^* (Iberian), *APP* ^E693G^ (Arctic)^[^ ^12a^ ^]^ mutation, and *MAPT* replacement from mouse tau to human tau^[^ ^12b^ ^]^ were kindly gifted from Prof. Takaomi C Saido (RIKEN BIO Co. Ltd.).^[^ ^32^ ^]^ *APP^NL‐G‐F^*/*MAPT* double knock‐in (dKI) mice were bred by crossing *APP* ^NL‐G‐F^ knock‐in mice and *MAPT* knock‐in mice. Mice were sacrificed at 12 and 18 months of age and perfused with PBS. Brain samples were collected and used for immunohistochemistry.

### Bulk RNA Sequencing Analysis

The preprocessed reads were aligned to GRCh38 using the STAR software (version 2.7.10a)^[^ ^33^ ^]^ and were saved in BAM format. To extract raw read counts per gene, FeatureCounts was used.^[^ ^34^ ^]^ R package DESeq2^[^ ^35^ ^]^ was utilized to perform differential gene expression analysis of the RNA‐seq data. The default Wald test was used for differential expression analysis and Benjamini–Hochberg method was applied to correct multiple tests. Genes with adjusted P‐values less than 0.05 were considered to be differentially expressed genes (DEGs). Protein‐protein network analysis was performed using the STRING database,^[^ ^36^ ^]^ and Gene Ontology (GO) analysis was performed using the Toppgene database.^[^ ^37^ ^]^

### Statistical Analyses

GraphPad Prism 8 (GraphPad Software, CA, USA) was appropriately used for all data analyses. Comparison of numerical data was performed using independent *t*‐test or ANOVA with post‐*hoc* analysis. Correlational significance was obtained via Pearson's correlation analysis. Live‐cell tracking was performed by using the TrackMate plugin^[^ ^38^ ^]^ in ImageJ (Fiji) software.^[^ ^39^ ^]^ Quantification of the microglial uptake was performed with Image J (Fiji) or IMARIS software.

## Conflict of Interest

The authors declare no conflict of interest.

## Author Contributions

J.‐C.P. and J.W.H. contributed equally to this work. J.‐C.P., J.W.H., I.M.‐J. performed conceptualization. J.‐C.P., J.W.H.,W.L., J.K., D.L., H.C., J.H., S.‐E.L., Y.J.K., Y.N.D., H.C., R.K., W.J.Y., J.L., M.C., S.‐W.I. performed investigation. J.‐C.P., J.W.H., W.L., S.‐E.L. performed methodology & visualization. J.‐C.P., W.L., S.E.L., J.‐I.K. performed resources. J.‐C.P., J.W.H., I.M.‐J. wrote‐the original draft. J.‐C.P., J.W.H., I.M.‐J. performed wrote‐review & edited the original draft.

## Supporting information

Supporting Information

Supplementary Video S1

Supplementary Video S2

Supplementary Video S3

Supplementary Video S4

Supplementary Video S5

Supplementary Video S6

Supplementary Video S7

Supplementary Video S8

## Data Availability

The data that support the findings of this study are openly available in NCBI GEO, GSE at https://www.ncbi.nlm.nih.gov/geo/query/acc.cgi?acc=GSE220690, reference number 220690.
