# Supplementary material for: Microglia Gravitate toward Amyloid Plaques Surrounded by Externalized Phosphatidylserine via TREM2
Source: Adv Sci (Weinh). 2024 Jul 9;11(34):2400064. doi: 10.1002/advs.202400064 (PMC11425970; doi:10.1002/advs.202400064)
Supplement: Supplementary file 1 — Supporting Information [file ADVS-11-2400064-s002.pdf]

ADVANCED SCIENCE Open Access

Supporting Information

for *Adv. Sci.*, DOI 10.1002/advs.202400064

Microglia Gravitate toward Amyloid Plaques Surrounded by Externalized Phosphatidylserine via TREM2

*Jong-Chan Park, Jong Won Han, Woochan Lee, Jieun Kim, Sang-Eun Lee, Dongjoon Lee, Hayoung Choi, Jihui Han, You Jung Kang, Yen N. Diep, Hansang Cho, Rian Kang, Won Jong Yu, Jean Lee, Murim Choi, Sun-Wha Im, Jong-Il Kim and Inhee Mook-Jung**

Supporting Information

Title: Microglia gravitate toward amyloid plaques surrounded by externalized phosphatidylserine via TREM2

Authors: Jong-Chan Park,[1,2,3,16] Jong Won Han,[4,16] Woochan Lee,[4,5] Jieun Kim,[4] Sang-Eun Lee,[6,7,8,9] Dongjoon Lee,[4] Hayoung Choi,[4] Jihui Han,[4] You Jung Kang,[1,2] Yen N. Diep,[1,2,10] Hansang Cho,[1,2,10] Rian Kang,[2,3] Won Jong Yu,[2,3] Jean Lee,[11] Murim Choi,[11] Sun-Wha Im,[12] Jong-Il Kim,[5,11,13,14] Inhee Mook-Jung,[4,15,17,*]

*Corresponding Author: Inhee Mook-Jung, Ph.D.

[17]Department of Biochemistry & Biomedical Sciences, Seoul National University College of Medicine, 103 Daehak-ro, Jongno-gu, Seoul 03080, Republic of Korea; Tel: +82-2-740-8245;

Fax: +82-2-3672-7352; Email: inhee@snu.ac.kr

[16]These authors contributed equally.

Supporting figures

Figure S1. Characterization of iPSC-derived microglia

Figure S2. Characterization of brain assembloids

Figure S3. Individual gene expressions and gating for cell-type annotations for single-cell RNA sequencing analysis.

Figure S4. Generation of iPSC-derived neurons and astrocytes

Figure S5. Comparison of microglial-specific genes in iMG, 2D co-cultured iMG, and 3D co-cultured iMG

Figure S6. Changes of microglia phenotypes in response to PtdSer treatment

Figure S7. Different microglial behavior toward ePtdSer$^+$Aβ

Figure S8. Comparison of microglial gene expressions between E3 iMG vs E4 iMG

Figure S9. Migration and cell death assay for TREM2 & APOE iMG lines

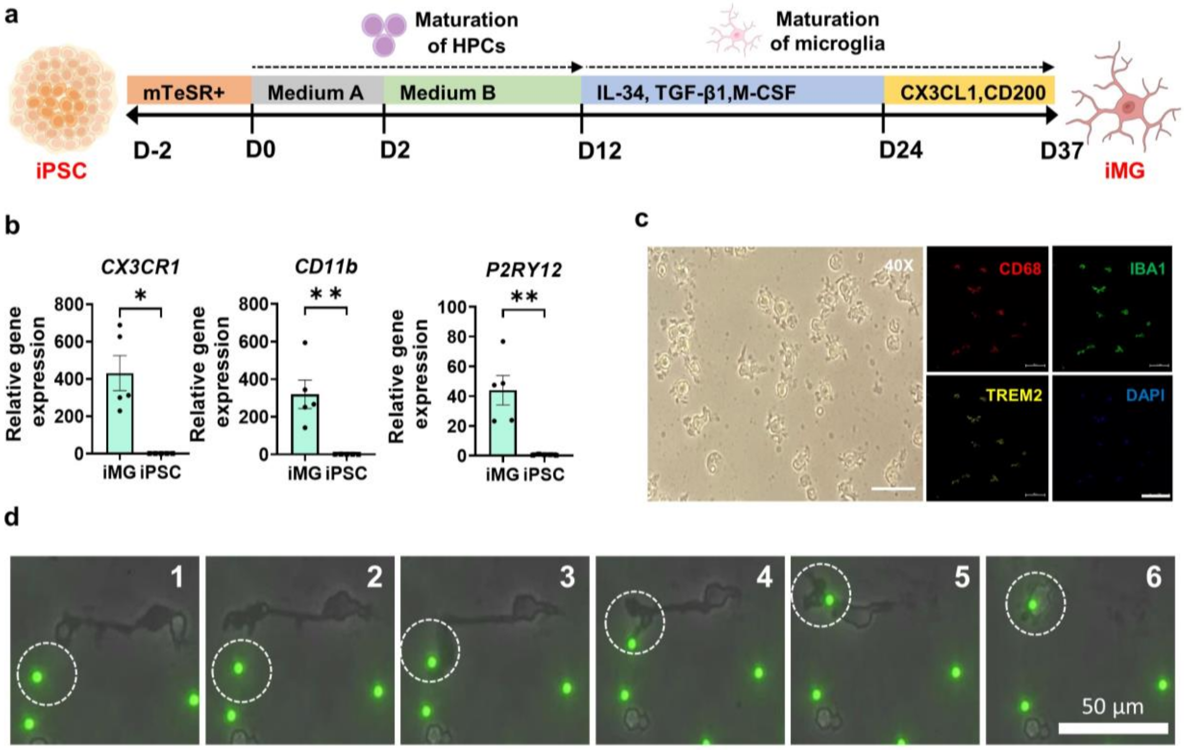

Figure S1. Characterization of iPSC-derived microglia. (a) A schematic diagram of iPSC-derived microglia (iMG) differentiation protocol. **(b)** iMG showed higher mRNA expression of microglia marker genes (*CX3CR1, CD11b, P2RY12*) than did iPSCs. H18 was used as a reference control. N = 5 includes three independent biological replicates (n = 3). *$p < 0.05$, **$p < 0.01$, independent *t*-test; ns, no significance. **(c)** Representative images of iMG at day 37. The iMG expressed microglia-specific markers such as CD68, Iba1, and TREM2 along with the nuclear staining DAPI. Red, anti-CD68; green, anti-Iba1; yellow, anti-TREM2; blue, DAPI. Scale bar = 50 µm. **(d)** Timelapse imaging for microglial uptake of GFP-fluorescence bead (white circle). green, GFP-fluorescence bead. Scale bar = 50 µm.

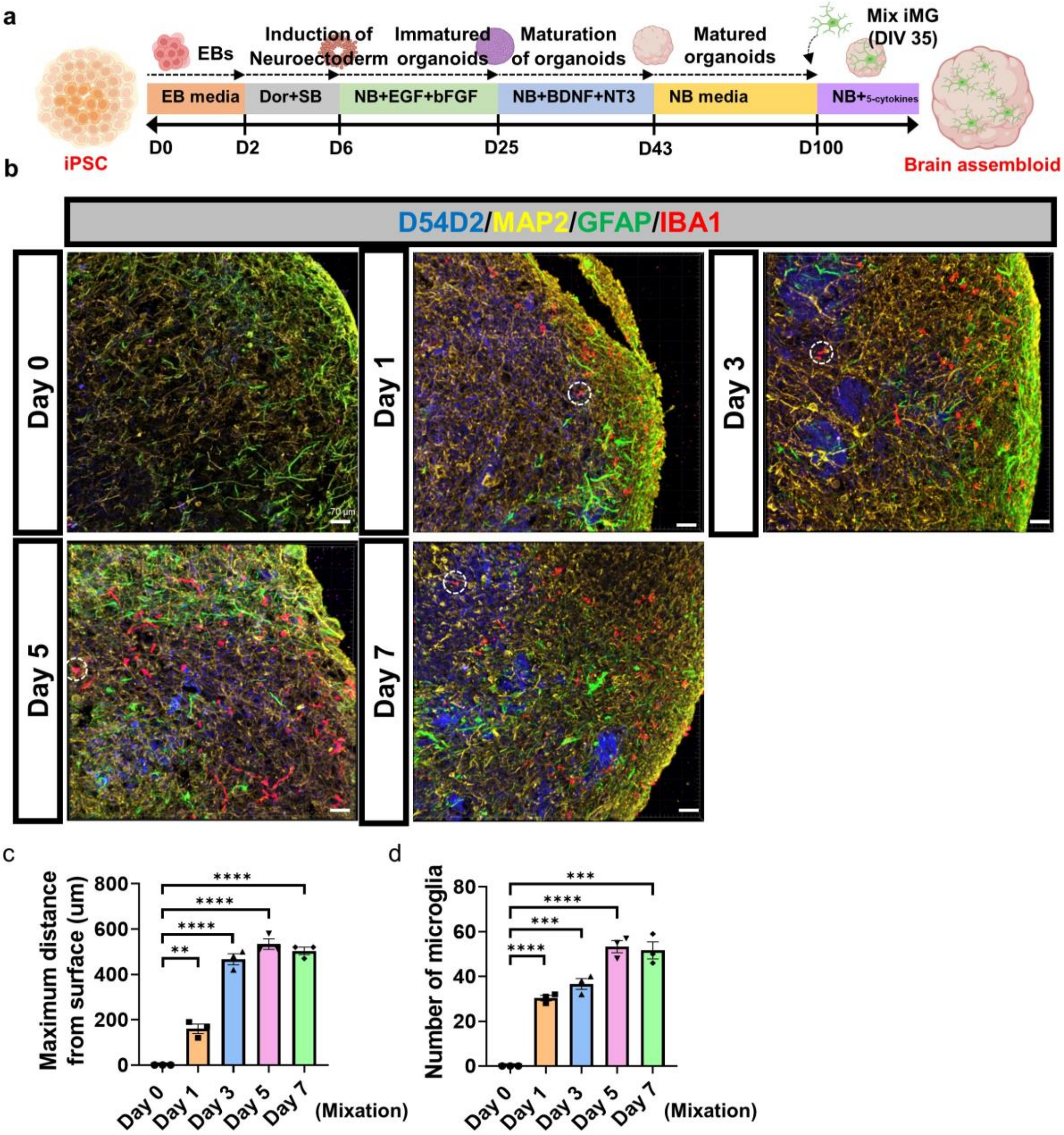

Figure S2. Characterization of brain assembloids. (a) A schematic diagram of brain assembloid generation protocol. **(b)** Representative immunostaining images of brain assembloids cultured on days 0, 3, 5, and 7. blue, anti-D54D2; green, anti-MAP2; yellow, anti-GFAP; red, Iba1. Scale bar = 70 µm. **(c)** Quantification of maximum distances of iMG from the surface after co-cultured with organoids on days 0, 3, 5, and 7. **$p < 0.01$ and ****$p < 0.0001$, independent *t*-test. (d) Quantification of the number of iMG after co-culture on days 0, 3, 5, and 7. The number of iMG gradually increased over time until day 5. ***$p < 0.001$ and ****$p < 0.0001$, independent *t*-test, sections for each day n = 3.

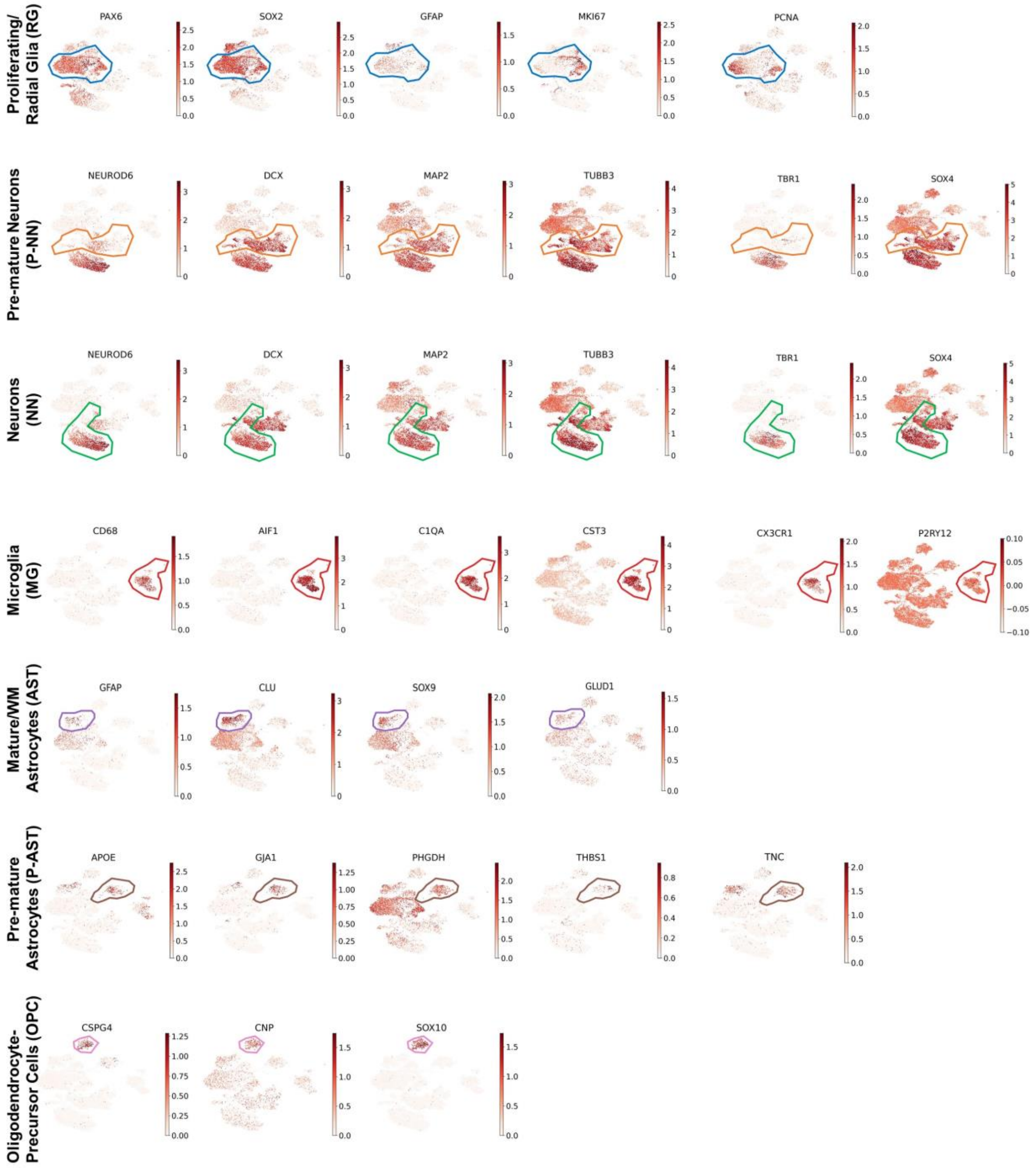

Figure S3. Individual gene expressions and gating for cell-type annotations for single-cell RNA sequencing analysis. All clusters are corresponded to Figure 1d. Each dot of cells is coloured according to their expression levels of genes.

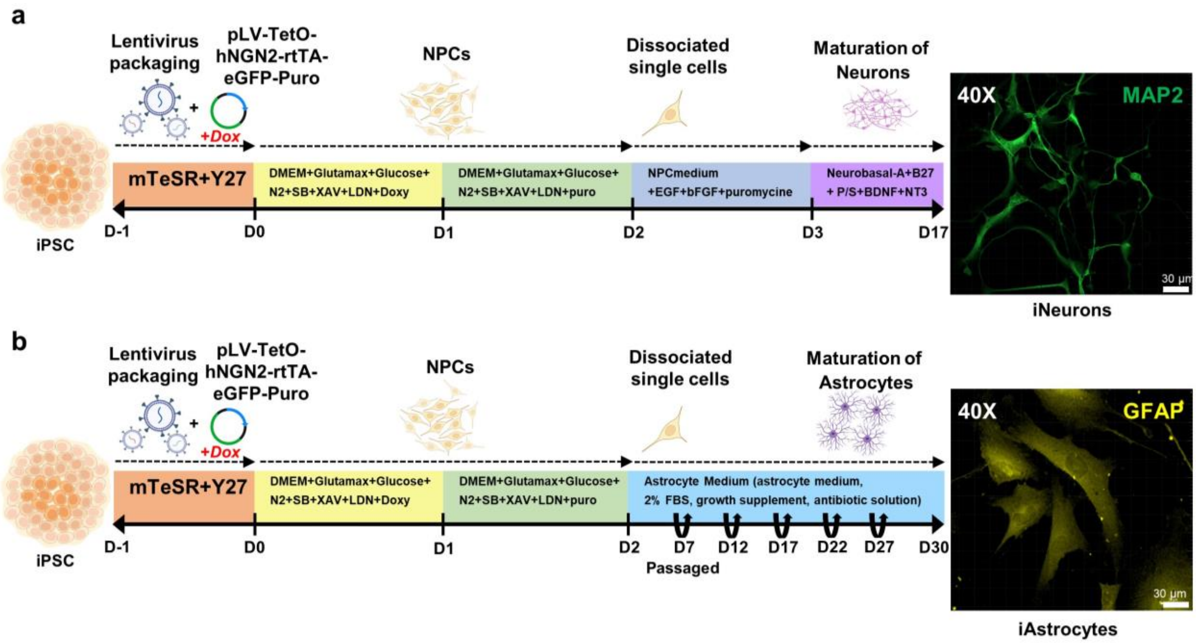

Figure S4. Generation of iPSC-derived neurons and astrocytes. (a) A schematic diagram of iPSC-derived neurons (iNeurons) differentiation protocol. green, anti-MAP2. Scale bar = 30 μm. **(b)** A schematic diagram of iPSC-derived astrocytes (iAstrocytes) differentiation protocol. yellow, anti-GFAP. Scale bar = 30 μm.

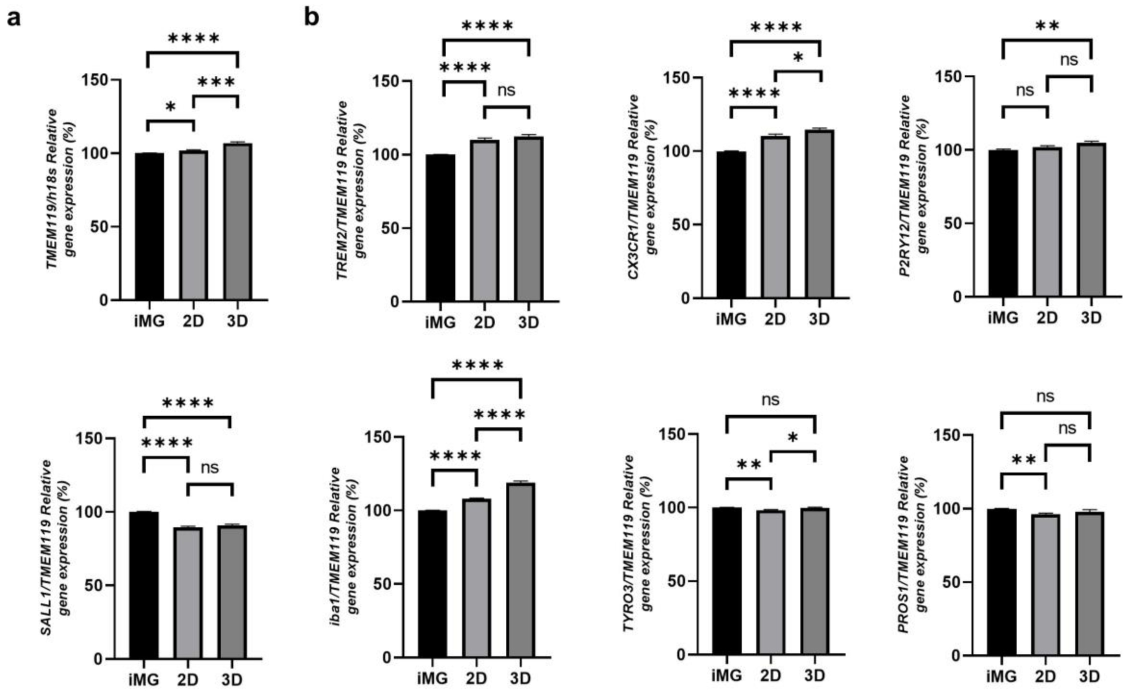

Figure S5. Comparison of microglial specific genes between in iMG, 2D co-cultured iMG, and 3D co-cultured iMG. (a) The 3D cultures consistently exhibited higher mRNA expression of the homeostatic microglial marker gene, TMEM119, compared to iMG and 2D cultures. H18 was utilized as the reference control. N = 3 represents three independent biological replicates (n = 3). *$p < 0.05$, ***$p < 0.001$ and ****$p < 0.0001$, independent *t*-test. **(b)** To ensure an accurate comparison of microglial-specific gene expression between iMG, 2D and 3D cultures, the microglial gene TMEM119 was used as a normalization reference. N = 3 represents three independent biological replicates (n = 3). *$p < 0.05$, **$p < 0.01$, ***$p < 0.001$ and ****$p < 0.0001$, independent *t*-test.

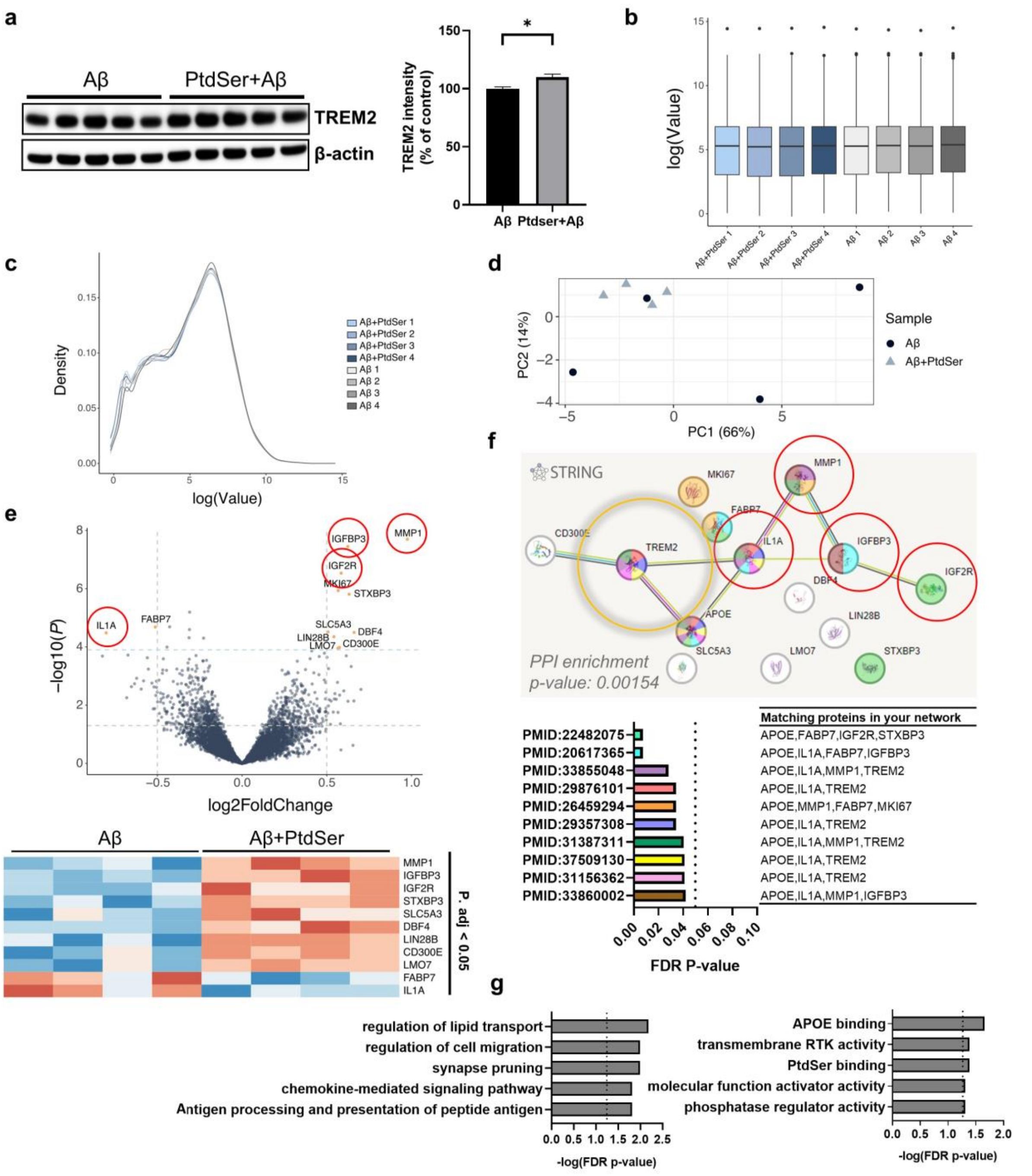

Figure S6. Changes of microglia phenotypes in response to PtdSer treatment. (a) Comparison of TREM2 levels between Aβ and Aβ+PtdSer groups. The Aβ+PtdSer group exhibited higher TREM2 protein expression levels compared to the Aβ+ group. *$p < 0.05$, independent *t*-test, each n = 5. **(b)** Gene expression distribution after normalization (box-plots with maximum 3th quartile+1.5IQR of upper whiskers, minimum 1th quartile -1.5IQR of lower whiskers, Q1 25th percentile hinge and Q3 75th percentile hinge for bounds of box, and Q2 50th percentile of median center bold line) from bulk RNA sequencing analysis (between Aβ vs Aβ+PtdSer groups). **(c)** Distribution pattern of gene expression. **(d)** Principal

component analysis (PCA) plot showing transcriptomic expression patterns in RNA sequencing data. **(e)** Volcano plot with the marks of Top-ranked genes (cut-off: log2(fold change) > 0.5 or < -0.5 and –log10(adjusted p-value) > 4. Red circles mean top-ranked genes according to both fold-change value and p-value. **(f)** Protein-protein interaction (PPI) results from the STRING database. Top-ranked genes are highly associated with TREM2. The relevant publications and their interaction scores are also presented as PMIDs and FDR-corrected p-values ($p < 0.05$). Interaction-matching proteins are also presented. **(g)** Gene ontology analysis using Toppgene database. Molecular Function (MF) (left graph) and Biological Process (right graph) terms were used. P-values obtained according to the Benjamini-Hochberg procedure FDR correction.

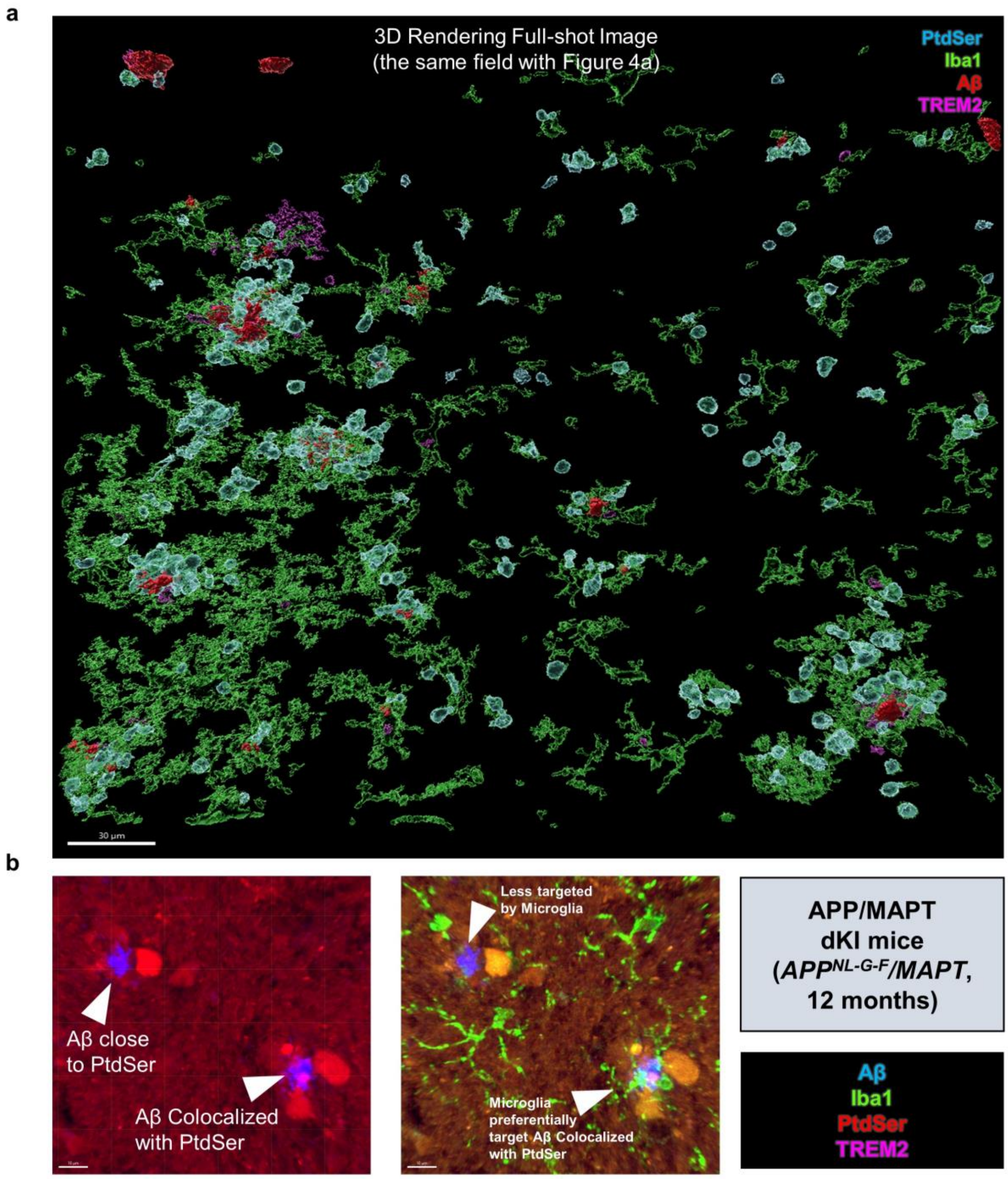

Figure S7. Different microglial behavior toward ePtdSer$^+$Aβ. (a) The 3D rendering image of Fig. 3c (full shot image). Scale bar = 30 μm. **(b)** Comparison of microglial behavior between Aβ without PtdSer and Aβ co-localized with PtdSer in *APP$^{NL\text{-}G\text{-}F}$ MAPT* dKI mice (12 months old). Aβ close to PtdSer was less targeted by microglia, but Aβ co-localized with PtdSer was preferentially targeted by microglia. blue, MX04; green, anti-Iba1; red, Ptdser; pink, anti-TREM2. Scale bar = 10 μm.

a

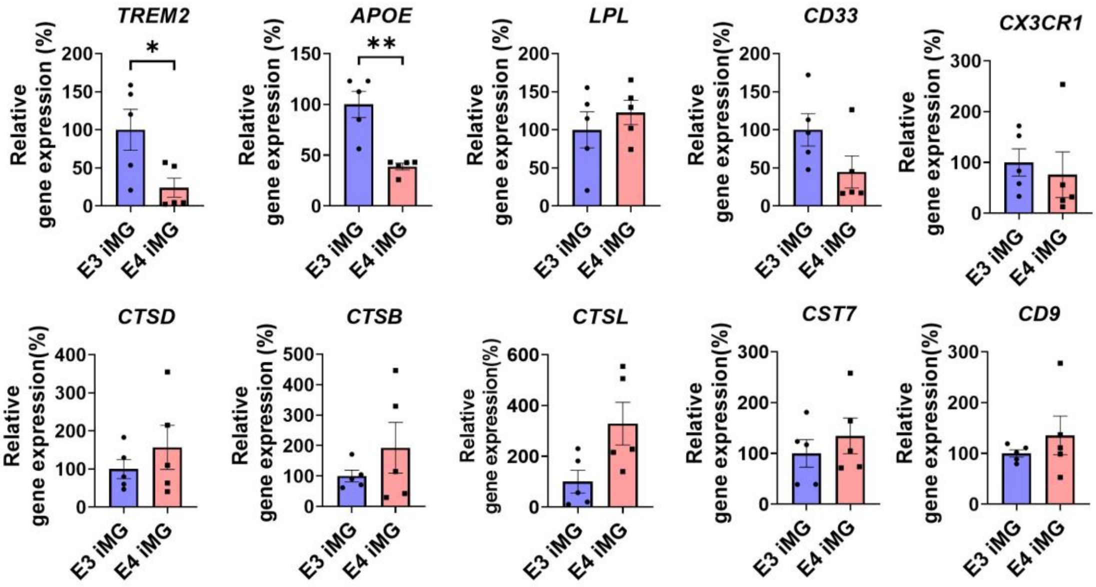

b

Gene	Forward primer (5'→3')	Reverse primer (5'→3')	Source/ Reference
**TREM2**	GGTCAGCACGCACAACTTG	CGCAGCGTAATGGTGAGAGT	PMID: 31665631
**APOE**	GTTGCTGGTCACATTCCTGG	GCAGGTAATCCCAAAAGCGAC	PMID: 32978453
**LPL**	CGAGCGCTCCATTCATCTCT	CCAGATTGTTGCAGCGGTTC	Confirmed in Primer-BLAST
**CD33**	GGCCACTCCAAAAACCTGAC	GACAACCAGGAGAAGATCGGG	PMID: 31208978
**CX3CR1**	ACTTTGAGTACGATGATTTGCT	GGTAAATGTCGGTGACACTCTT	PrimerBank ID: 283837933c1
**P2RY12**	CACTGCTCTACACTGTCCTGT	AGTGGTCCTGTTCCCAGTTTG	PMID: 35069843
**CD11b**	GGAACGCCATTGTCTGCTTTCG	ATGCTGAGGTCATCCTGGCAGA	PMID: 35712074
**CTSD**	TGCTCAAGAACTACATGGACGC	CGAAGACGACTGTGAAGCACT	PMID: 29025968
**CTSB**	GAGCTGGTCAACTATGTCAACA	GCTCATGTCCACGTTGTAGAAGT	PMID: 35392078
**CST7**	GTGTGAAGCCAGGATTTCCTAA	TGTCGTTCGTGCAGTTGTTGA	PMID: 28583823
**CTSL**	CTTTTGCCTGGGAATTGCCTC	CATCGCCTTCCACTTGGTC	PMID: 34091590
**SALL1**	GCTTGCACTATTTGTGGAAGAGC	GAACTTGACGGGATTGCCTCCT	Confirmed in Primer-BLAST
**PROS1**	GGCTCCTACTATCCTGGTTCTG	CAAGGCAAGCATAACACCAGTGC	Confirmed in Primer-BLAST
**TMEM119**	GGATAGTGGACTTCTTCCGCCA	GGAAGGACGATGGGTAATAGGC	Confirmed in Primer-BLAST
**TYRO3**	GCA AGC CTT TGA CAG TGT CAT GG	GTT CAT CGC TGA TGC CCA AGC T	Confirmed in Primer-BLAST
**h18s**	GGCCCTGTAATTGGAATGAGTC	CCAAGATCCAACTACGAGCTT	PMID: 30712844

Figure S8. Comparison of microglial gene expressions between E3 iMG vs E4 iMG. (a) Comparison of mRNA expression levels of microglial genes between ApoE ε3/ε3 and ε4/ε4 iMG. $^{**}p < 0.01$, $^{*}p < 0.05$, independent *t*-test. **(b)** List of primers used in the present study.

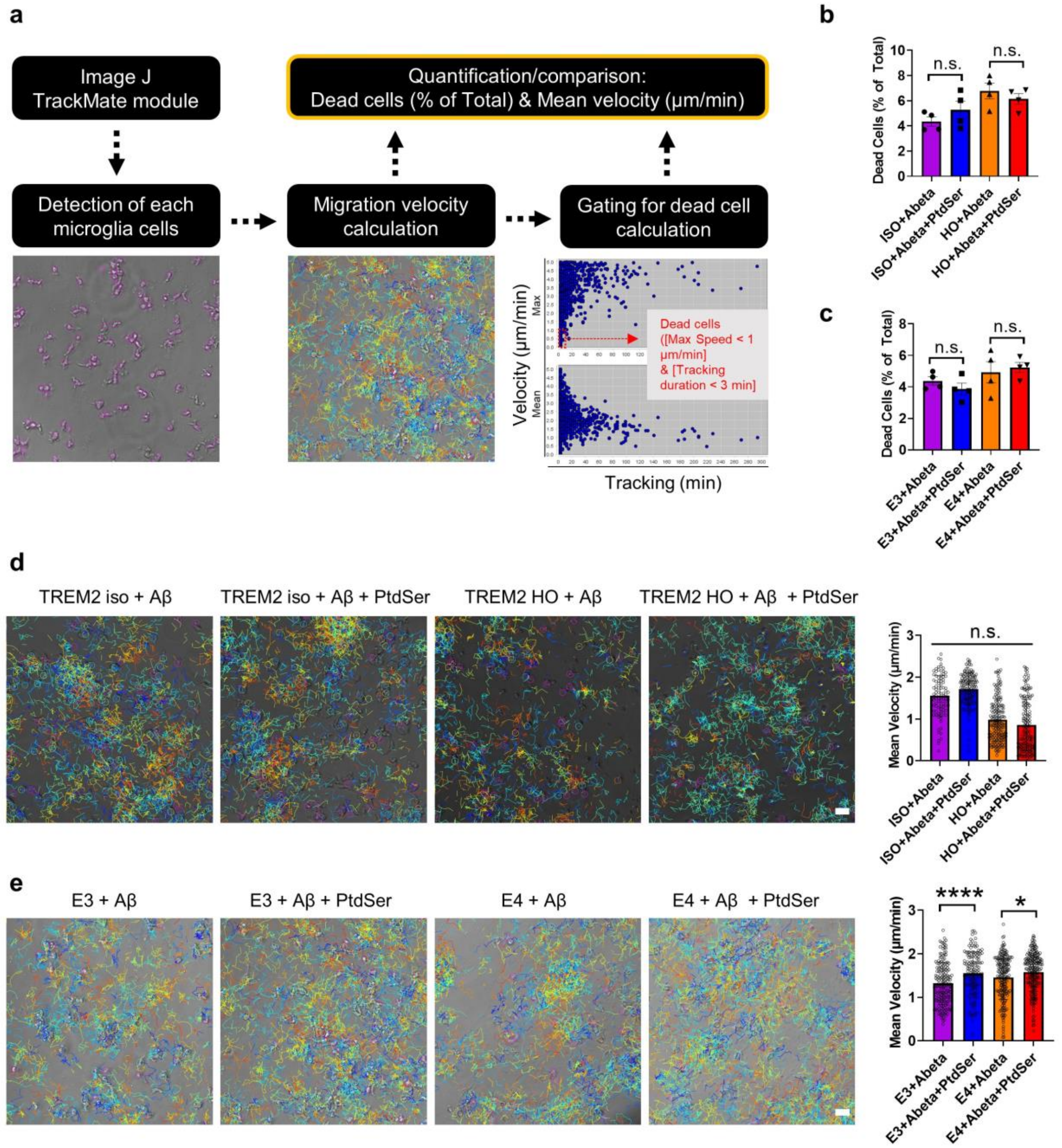

Figure S9. Migration and cell death assay for TREM2 & APOE iMG lines. (a) Overall TrackMate workflow for tracking iMG migration and cell death. Migration velocity was calculated as mean values (μm/min), and the number of dead cells were calculated with the following criteria: [Max speed < 1 μm/min] and [Tracking duration < 3 min]. **(b-c)** Comparison of cell viability between the groups for both TREM2 and APOE iMG lines. **(d-e)** Comparison of migration mean velocity between TREM2 iMG lines and APOE iMG lines. n.s., non-significant results; p-values by ANOVA with post-hoc test; HO, TREM2 homozygous mutation line; ISO, isogenic control; Abeta, beta-amyloid; PtdSer, phosphatidylserine. *$p < 0.05$ and ****$p < 0.0001$; Two-way ANOVA with post-hoc; N = 6

slides for each group; average n = 114.93 cells were tracked per one field of view (scale bar = 30 μm).
